# A Human Engineered Heart Tissue‐Derived Lipotoxic Diabetic Cardiomyopathy Model Revealed Early Benefits of Empagliflozin

**DOI:** 10.1002/advs.202503173

**Published:** 2025-05-28

**Authors:** Lin Cai, Yuxin Zhao, Zilong Li, Liping Xiao, Yifan Wu, Shiya Wang, Qian Liu, Yida Ye, Yuxuan Guo, Donghui Zhang

**Affiliations:** ^1^ State Key Laboratory of Biocatalysis and Enzyme Engineering Stem Cells and Tissue Engineering Manufacture Center School of Life Sciences Hubei University Wuhan 430062 China; ^2^ Key Laboratory of Animal Biological Products & Genetic Engineering Ministry of Agriculture and Rural Sinopharm Animal Health Corporation Ltd. Wuhan 430023 China; ^3^ Institute of Cardiovascular Sciences School of Basic Medical Sciences State Key Laboratory of Vascular Homeostasis and Remodeling Beijing Key Laboratory of Cardiovascular Receptors Research Peking University Beijing 100191 China

**Keywords:** diabetic cardiomyopathy, diastolic dysfunction, empagliflozin, hEHT, in vitro model

## Abstract

Diabetic cardiomyopathy (DbCM) is increasingly prevalent, but intervention targets remain unclear due to the lack of appropriate models and the complexity of risk factors. Here, this work establishes an in vitro assessment system for DbCM function using cardiomyocytes derived from human pluripotent stem cells and engineered heart tissue. This work finds high‐fat status in complex diabetes risk factors majorly contributes most to cardiomyocyte death and contractile dysfunction. Notably, PA induced early electrophysiological abnormalities, and lately is associated with cardiac fibrosis, mitochondrial fission, and systolic and diastolic dysfunction at tissue level. Using this in vitro assessment system, this work finds that empagliflozin (EMPA), a first‐line glucose‐lowering drug, effectively alleviated early PA‐induced cardiomyocyte injury. Treatment with EMPA enhanced abnormal diastolic and electrophysiological functions in the PA‐hEHT model and significantly reduced endoplasmic reticulum stress, and apoptosis. Furthermore, these promising results are confirmed in a type 2 diabetes mellitus mouse model, reinforcing the potential of EMPA as a therapeutic option to alleviate cardiomyocyte injury under diabetic conditions. These findings suggest that this work has developed an engineered model of diabetic cardiomyopathy that mimics the various stages of lipotoxic myocardial injury and support the use of EMPA as a potential therapeutic option for diabetic or lipotoxic cardiomyopathy.

## Graphical abstract

This study develops a human engineered heart tissue‐derived model of diabetic cardiomyopathy that accurately replicates the dynamic changes in the structural, contractile, and electrophysiological properties of the myocardium. Furthermore, this model is used to confirm the direct protective effects of empagliflozin on the heart through an SGLT2 off‐target mechanism, consistent with findings from mouse models.

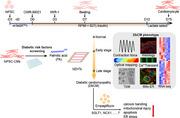

## 1 Introduction

Diabetes has emerged as a significant global health challenge,^[^ ^1^ ^]^ with China being particularly affected by this epidemic, where ≈11.2% of the adult population is diagnosed with diabetes.^[^ ^2^ ^]^ Cardiovascular diseases represent a leading cause of morbidity and mortality among individuals with type 2 diabetes mellitus (T2DM), with heart failure (HF) frequently manifesting as a common complication.^[^ ^3^, ^4^, ^5^ ^]^ Among the various complications of diabetes, diabetic cardiomyopathy (DbCM) stands out as the most prevalent. Initially characterized as a distinct form of cardiomyopathy marked by cardiac hypertrophy, fibrosis, structural and functional impairment independent of preexisting cardiovascular risk factors such as hypertension or coronary artery disease,^[^ ^6^ ^]^ DbCM has recently been redefined to encompass systolic and/or diastolic myocardial dysfunction occurring in the context of diabetes—an entity referred to as diabetic myocardial disorder.^[^ ^7^ ^]^ This revised definition broadens the scope of DbCM, placing greater emphasis on myocardial function and highlighting the direct myocardial damage experienced by individuals with diabetes.

DbCM/diabetic myocardial disorder presents a series of characteristics associated with multiple mechanisms that remain incompletely elucidated.^[^ ^7^, ^8^ ^]^ One significant challenge in elucidating these mechanisms is the involvement of complex systemic factors, including hyperglycemia, insulin resistance, hyperlipidemia, advanced glycation end products, renin‐angiotensin‐aldosterone system activation, and autonomic dysregulation, which interrelate to cause the development of diabetes, hypertension, accelerated coronary atherosclerosis, and microvascular dysfunction.^[^ ^9^, ^10^, ^11^ ^]^ Numerous studies have shown that they jointly lead to abnormal myocardial metabolism, glucotoxicity, and lipotoxicity, ultimately leading to oxidative stress, fibrosis, hypertrophy, diastolic dysfunction, and systolic dysfunction.^[^ ^12^, ^13^ ^]^ However, it is challenging to analyze these factors separately. Additionally, a critical obstacle in managing DbCM is the early detection of diastolic dysfunction, which is often obscured by subtle symptoms.^[^ ^14^, ^15^ ^]^ By the time both systolic and diastolic dysfunction deteriorate, the complexity of intervention increases significantly and the prognosis becomes increasingly poor.^[^ ^16^ ^]^ Therefore, it is essential to develop dynamic models that encompass the diverse subtypes of T2DM patients.^[^ ^17^ ^]^ These models should be based on stratifying risk factors and understanding the evolving pathophysiological changes from early to late stages. Such an approach is imperative for creating effective prevention and treatment strategies for heart failure within the T2DM population.

Experimental models, including both animal models and in vitro cell models, have provided significant mechanistic and phenotypic insights into the pathogenesis underlying DbCM.^[^ ^18^, ^19^ ^]^ Transgenic, drug‐induced, and high‐glucose/high‐fat animal models have greatly enhanced our understanding of DbCM pathophysiology, including mitochondrial dysfunction, impaired Ca^2+^ handling, ER stress and cell death.^[^ ^17^, ^18^, ^19^, ^20^ ^]^ However, limitations such as species‐specific genetic backgrounds and long experimental schedules persist in these models. Primary adult human cardiomyocytes are optimal for in vitro studies, but they face challenges related to sample scarcity and difficulties in culture. In this context, human induced pluripotent stem cell (hiPSC)‐derived cardiomyocytes (hiPSC‐CMs) present a promising alternative, providing a substantial number of seed cells for phenotypic modeling and risk factor assessment in DbCM research.^[^ ^21^, ^22^, ^23^ ^]^ However, iPSC‐CMs exhibit structural, metabolic, and electrophysiological immaturity compared to adult cardiomyocytes.^[^ ^24^, ^25^ ^]^ These immature properties can be improved through long‐term culture (up to 360 days),^[^ ^26^ ^]^ biochemical factors, mechanical strain, or electrical stimulation.^[^ ^24^, ^27^, ^28^, ^29^ ^]^ Meanwhile, 3D culture systems such as engineered heart tissues (EHTs) and cardiac organoids have demonstrated superior maturation of hiPSC‐CMs compared to traditional 2D cultures,^[^ ^30^, ^31^ ^]^ and are now widely used to model various cardiomyopathies and for drug screening applications,^[^ ^32^, ^33^, ^34^ ^]^ including those related to DbCM.^[^ ^35^, ^36^ ^]^ Using an organoid system, researchers have successfully modeled embryonic heart development under pregestational diabetes‐like conditions, revealing ROS‐mediated ER stress and abnormal lipid metabolism.^[^ ^37^ ^]^ Another recently study evaluated the cardiotoxic effects induced by a high‐glucose and high‐lipid environment, including apoptosis, oxidative stress, and mitochondrial dysfunction, in spherical cardiac organoids.^[^ ^38^ ^]^ However, none of these current models comprehensively address the dynamic alterations in contractile and electrophysiological properties characteristic of diabetic hearts.^[^ ^17^ ^]^ Spheroid organoids rely on self‐aggregation through spontaneous co‐differentiation of cells in 3D aggregates, while hEHTs are constructed by embedding hiPSC‐CMs within a hydrogel scaffold to form bundles, patches, or other structures according to different needs, which provides relatively better structural and/or electromechanical maturation compared to spheroids.^[^ ^39^, ^40^, ^41^, ^42^ ^]^ Although the long‐term stability of hEHTs can be further improved through more flexible technologies and compatible hydrogels, the hEHT‐based model offers a promising and adaptable platform for modeling the dynamic changes in contractile and electrical properties of DbCM, facilitating further drug screening, verifying the efficacy and mechanism of potential drugs, and serving as an important supplement to animal models.

Sodium‐glucose cotransporter‐2 inhibitors (SGLT‐2is) are a class of prescription medications approved to lower blood sugar in adults with type 2 diabetes (T2DM) when combined with diet and exercise.^[^ ^43^ ^]^ Beyond glycemic control, SGLT2is, including dapagliflozin and empagliflozin (EMPA), have demonstrated benefits in managing heart failure.^[^ ^44^, ^45^ ^]^ They reduce the risk of cardiovascular hospitalization and death in patients with HFrEF (heart failure with reduced ejection fraction)^[^ ^46^ ^]^ and lower the risk of cardiovascular and all‐cause mortality in those with HFpEF (heart failure with preserved ejection fraction).^[^ ^47^, ^48^ ^]^ However, whether SGLT2 inhibitors exert direct effects on the heart or act solely through systemic improvements remain controversial, and the underlying mechanisms of SGLT2i‐mediated cardioprotection continue to be a significant topic of interest in the field. On one hand, beyond their antihyperglycemic effects, SGLT2 inhibitors such as EMPA exhibits osmotic diuretic and natriuretic properties that contribute to improvements in cardiovascular and kidney diseases.^[^ ^49^ ^]^ Additionally, EMPA has been shown to reduce the formation of uremic toxins by modulating the microbiome, as demonstrated through comprehensive proteomics, phosphoproteomics, and metabolomics analyses.^[^ ^50^ ^]^ On the other hand, although SGLT2 is primarily expressed in the kidney and is virtually absent in the human heart, growing evidence indicates that SGLT2 inhibitors can directly inhibit sodium‐loaders such as sodium‐hydrogen antiporter 1 (NHE ‐1), voltage‐gated sodium channel Nav1.5, SGLT1, thereby exerting direct cardiac effects.^[^ ^51^, ^52^, ^53^ ^]^ Despite these promising findings, SGLT2 inhibitors remain underutilized in many eligible patients.^[^ ^54^ ^]^ Therefore, further studies employing diverse experimental systems or models are needed to clarify these controversies and provide new insights.

To establish a research platform for investigating the dynamic changes in contractility associated with DbCM, this study employed hEHT to assess the detrimental effects of risk factors on the myocardium. Specifically, we examined the role of excessive palmitic acid (PA) in inducing DbCM. By integrating contractility, tissue structure, electrophysiology, and RNA sequencing analyses, we sought to elucidate the underlying biological processes contributing to the progression of DbCM from early diastolic impairment to systolic and diastolic dysfunction. Moreover, we investigated the therapeutic potential of empagliflozin, a first‐line diabetes medication, in iPSC‐CMs and hEHT models, providing direct evidence of its cardioprotective role and exploring the underlying mechanisms. Finally, we validated these findings in a T2DM mouse model.

## 2 Results

### 2.1 Identification of Palmitic Acid as a Representative Diabetic Pathogenic Factor of Cardiac Injury

**Figure 1:**
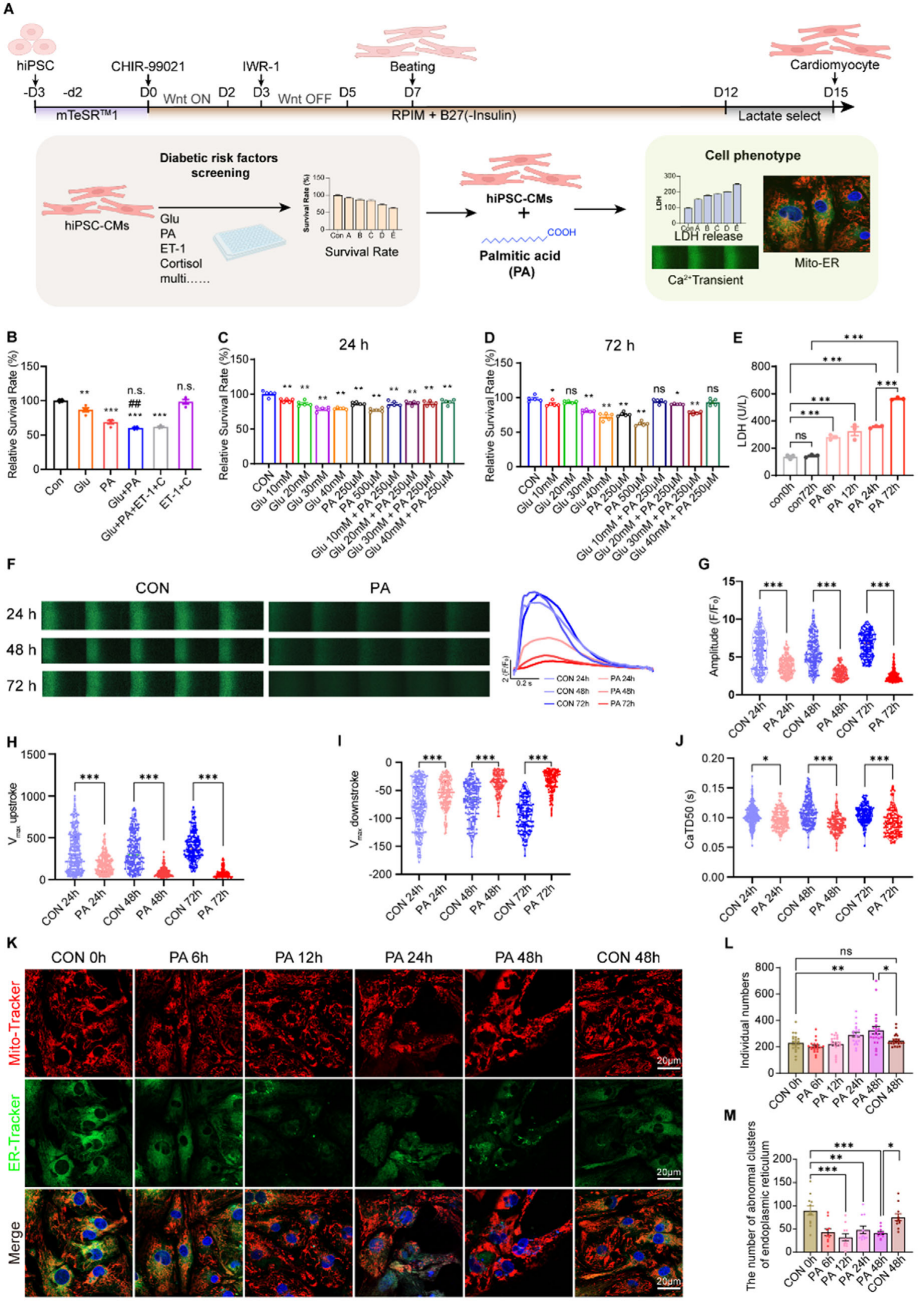
Palmitate acid represents a key diabetogenic risk factor that induces injury in iPSC‐CM. A) Scheme for evaluating the effects of diabetes risk factors on iPSC‐CMs. B–D) CCK8 assay showing survival of iPSC‐CM treated‐with high glucose (Glu), palmitic acid (PA), endothelin‐1(ET‐1) and cortisol (C) alone or in combination (B), or different concentrations of Glu or PA for 24 h (C) and 72 h (D) (n = 5). E) Lactate dehydrogenase (LDH) release from iPSC‐CM with PA treatment for the indicated time points. (n = 3). F–J) Representative images (F) and quantification (G) of Ca^2+^ transient in PA‐treated iPSC‐CMs for 24 h/48 h/72 h and the indicated control. iPSC‐CMs were loaded with Fluo‐4 AM and paced at 1 Hz, amplitude (H), maximum upstroke velocity (I), and duration at 50% repolarization (CaTD50) (J) were analyzed. K–M) Representative confocal images (K) and quantitative analysis of live Mito‐Tracker (L) and ER‐Tracker (M) staining in reseeded iPSC‐CMs at the indicated time points. (n = 8 images per group). ns *p* > 0.05, * *p* < 0.05, ** *p* < 0.01, *** *p* < 0.001, ^##^ *p* < 0.01 versus PA group in (B).

Myocardial structural and functional damage in diabetic cardiomyopathy is primarily attributed to cardiomyocyte dysfunction and is influenced by various diabetes risk factors.^[^ ^55^ ^]^ To assess the relative impact of these risk factors, we utilized a CCK‐8 assay on iPSC‐CMs, as shown in the strategy flow chart in **Figure** **1**A. The evaluated factors included high glucose, high fatty acid (palmitic acid, PA), endothelin 1 and cortisol^[^ ^23^ ^]^ alone or in combination. By implementing a step‐wise modulation of Wnt signaling, we achieved a high‐purity iPSC‐derived cardiomyocyte (iPSC‐CM) population exceeding 80% purity (Figure S1A–C, Supporting Information). Our findings revealed that PA exerted the most pronounced effect, significantly reducing cardiomyocyte viability (Figure 1B), particularly in synergy with varying glucose concentrations, mimicking distinct diabetic conditions (Figure 1C,D). Given the established correlation between serum free fatty acid levels and the severity of T2DM,^[^ ^56^, ^57^ ^]^ as well as our observed dose‐dependent results (Figure S2, Supporting Information), we selected 500 µM PA as the stimulating condition, which induced a time‐dependent increase in the release of lactate dehydrogenase (LDH), a recognized marker of myocardial injury, as shown in Figure 1E. Moreover, calcium transient analysis was assessed to evaluate systolic and diastolic function. Compared to controls, PA‐treated cardiomyocytes exhibited a progressive decrease in amplitude starting at 24 h (Figure 1F,G), accompanied by reduced calcium release and reuptake rates, and shortened 50% of Ca^2+^ transient duration (CaTD50) (Figure 1H–J), indicative of impaired systolic and diastolic function. Considering the well‐documented roles of mitochondrial dysfunction and ER stress in diabetic cardiomyopathy,^[^ ^8^ ^]^ we investigated mitochondrial and ER morphology within 48 h of PA treatment. Mitochondrial content transiently increased at 12 h before declining, coinciding with a gradual rise in mitochondrial fission (Figure 1K,L), resembling increased mitochondrial fission observed in DCM patients. Additionally, the endoplasmic reticulum exhibited abnormal aggregation and reduced overall quantity between 12‐ and 48‐h post‐PA treatment (Figure 1M). Altogether, these results indicated PA as a crucial pathogenic factor that impaired both cardiomyocyte function and viability.

### 2.2 Palmitic Acid Induced Diastolic Dysfunction in Human Engineered Heart Tissues in Short Term

**Figure 2:**
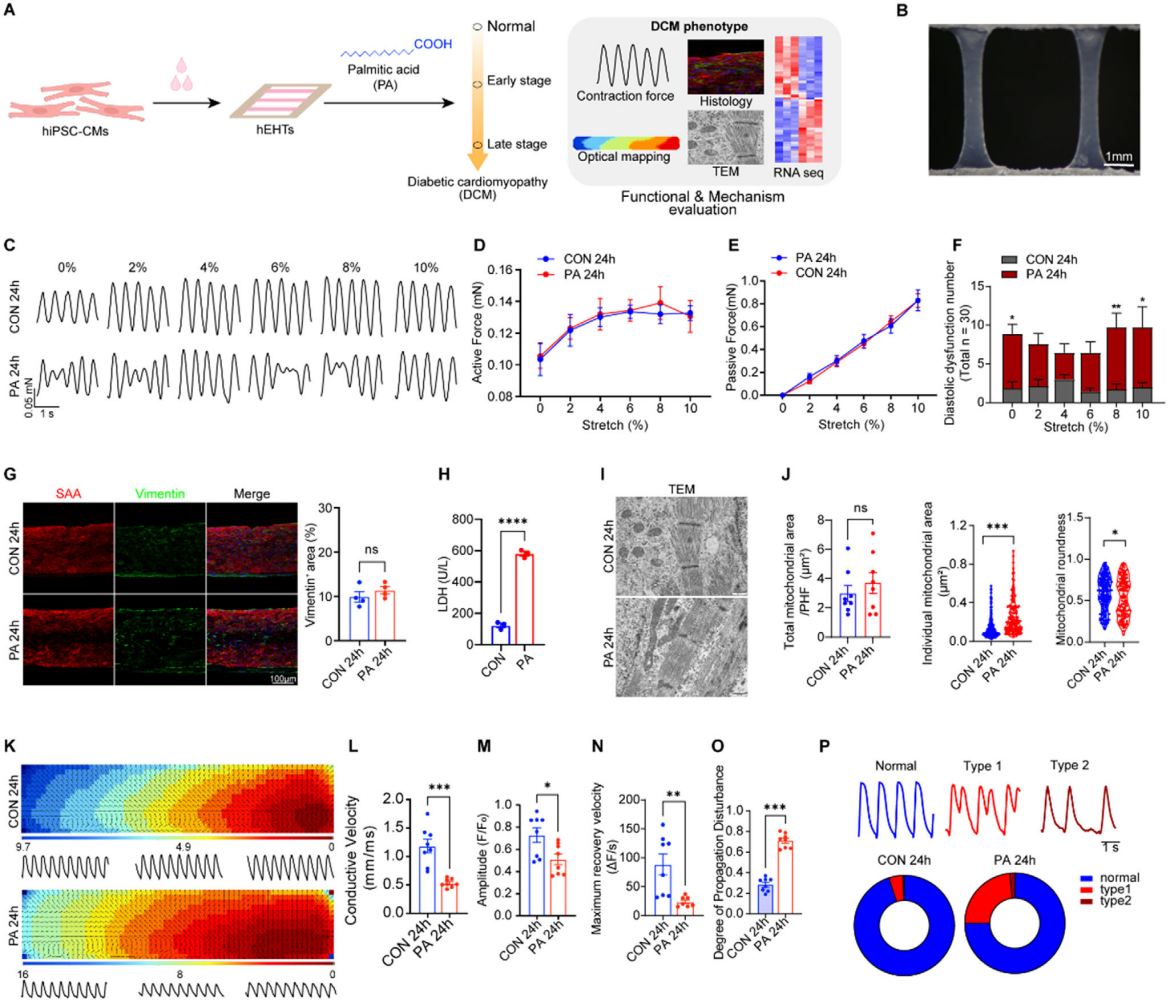
PA‐induced hEHT models of early diabetic cardiomyopathy injury. A) Scheme for evaluating the early and late effects of PA on hEHT. B) Physical image of hEHT on day 7. Scale bar = 1 mm. C–F) Schematic diagram (C) and statistical analysis of characteristic peaks, including active contractile force (D), passive contractile force (E), and the number of diastolic dysfunction peaks (F) under different stretching conditions of hEHT during progressive stretching with electrical stimulation (1.5 Hz) after 24 h of treatment in PA stimulation (n = 7 per group). G) Immunofluorescence staining images (left) and quantification (right) of the percentage of Vimentin^+^ cells in the hEHTs at 24 h post‐PA treatment. Scale bar = 100 µm. H) Lactate dehydrogenase (LDH) release from hEHTs following 24 h of PA treatment. (n = 4). I,J) Transmission electron microscopy (TEM) images showing the morphology of mitochondria and sarcomeres in hEHTs from both the PA and control groups (I), along with quantitative analysis of total mitochondrial area, individual mitochondrial area, and mitochondrial roundness (J). Scale bar = 500 nm. K–P) Optical mapping of hEHTs in the PA and control groups at 24 h, stimulated at a pacing frequency of 1 Hz. The results included isochronal activation and propagation direction maps alongside calcium transient traces (K), as well as statistical analysis of Ca^2^⁺ fluorescence signal conduction velocity (L), amplitude (M), maximum downstroke velocity (N), propagation direction dispersion (O), and the frequency distribution of two different types of diastolic dysfunction occurrences (P). n = 8 for the control group, n = 7 for the PA group. ns *p* > 0.05, * *p* < 0.05, ** *p* < 0.01, *** *p* < 0.001, **** *p* < 0.0001.

Human engineered heart tissue represents an ideal option for cardiomyopathy modeling.^[^ ^58^ ^]^ In this study, hiPSC‐derived EHTs were utilized for tissue‐level phenotype assessment (**Figure** **2**A). As approximately one quarter of Type 2 diabetic individuals exhibit asymptomatic myocardial structural and functional abnormalities, with the majority presenting diastolic dysfunction.^[^ ^14^ ^]^ Developing in vitro models to mimic diastolic dysfunction is crucial for studying diabetic cardiomyopathy. To dynamically evaluate changes in structure and contraction force, we designed a mold to construct 6 mm × 3 mm bundle‐shaped hEHTs (Figure 2B, Figure S3A,B, Supporting Information). Using a custom‐built mechanical sensing and stretching platform, we investigated the early effects of PA on hEHT function while preserving normal systolic parameters. Although 24 h of PA stimulation did not significantly alter baseline or stretch‐induced active and passive forces (Figure 2C–E), it induced subtle reductions in contraction and relaxation Vmax (Figure S4A,B, Supporting Information). Moreover, a notable increase in abnormal twin‐peak contractions under various stretch conditions in the PA group indicated impaired diastolic filling (Figure 2F), corroborating the development of early diastolic dysfunction.

While immunofluorescence staining for vimentin did not reveal significant alterations in cell type and fibrosis (Figure 2G), increased LDH release (Figure 2H) indicated PA‐induced injury in hEHTs. Moreover, transmission electron microscopy (TEM) revealed an increase in individual mitochondrial area and a decrease in mitochondrial roundness (Figure 2I,J), indicative of morphological changes in mitochondrial fusion. We further assessed the electrophysiological characteristics of hEHTs using optical mapping. PA stimulation significantly reduced electrical conduction velocity, amplitude, and related parameters (Figure 2K–O). Moreover, the frequency of abnormal contraction peaks associated with diastolic dysfunction was markedly increased (Figure 2P). Altogether, these findings demonstrate that a short‐term exposure (24 h) to PA induces early diastolic dysfunction in hEHT without compromising systolic function. These changes are accompanied by subcellular structural alterations indicative of incipient hypertrophy and electrical conduction abnormalities.

### 2.3 Prolonged PA Stimulation Recapitulates Systolic Dysfunction Features of Diabetic Cardiomyopathy in hEHTs

**Figure 3:**
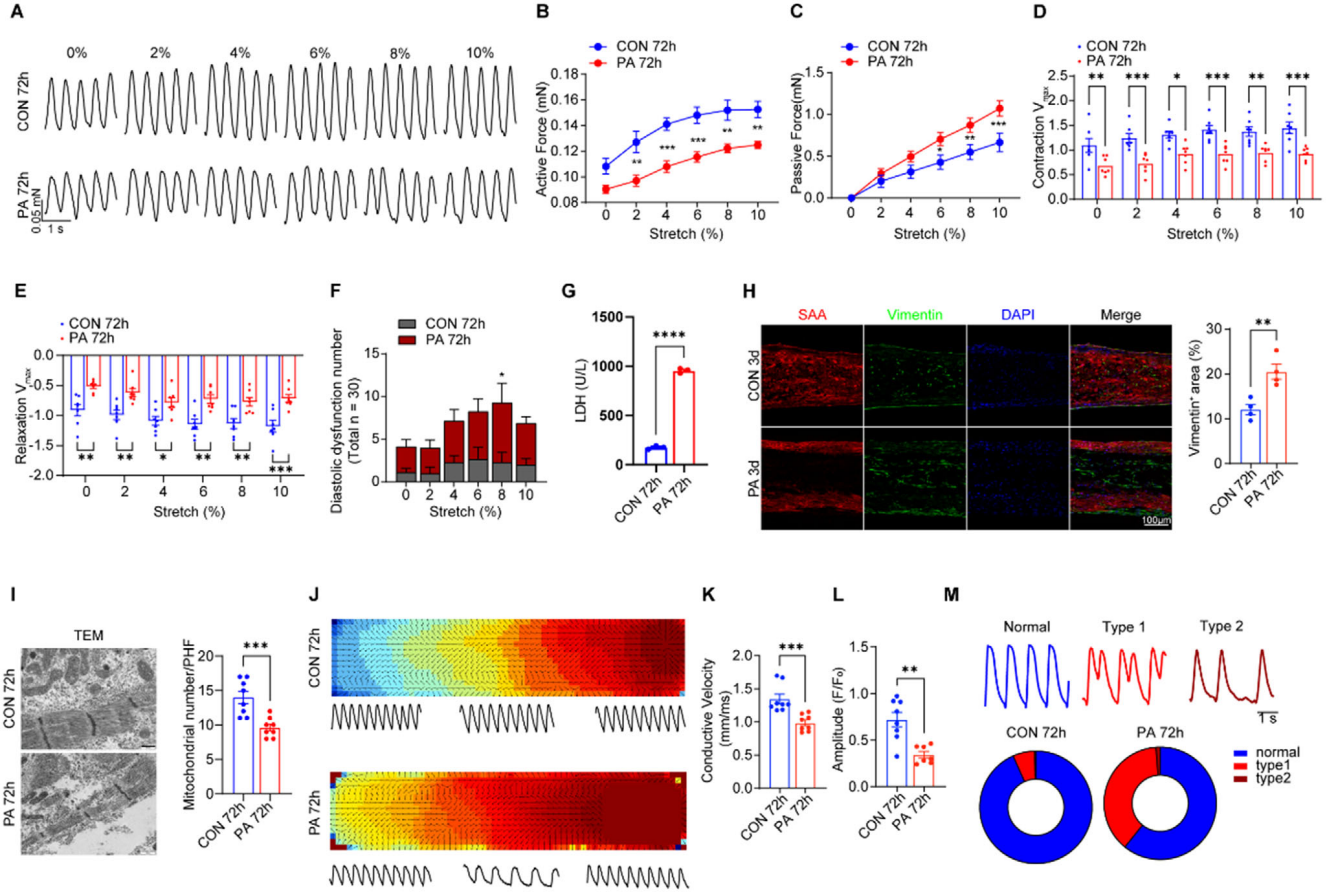
PA‐induced hEHT models of later diabetic cardiomyopathy injury. A–F) Schematic diagram (A) and statistical analysis of characteristic peaks, including active contractile force (B), passive contractile force (C), maximum contraction velocity (D), maximum relaxation velocity (E), and the number of diastolic dysfunction peaks (F) under different stretching conditions of hEHT during progressive stretching with electrical stimulation (1.5 Hz) after 72 h of treatment in PA stimulation (n = 7 per group). G) Lactate dehydrogenase (LDH) release from hEHTs following 72 h of PA treatment. (n = 3). H) Immunofluorescence staining images (left) and quantification (right) of the percentage of Vimentin^+^ cells in the hEHTs at 72 h post‐PA treatment. Scale bar = 100 µm. I) Representative TEM images and quantitative analysis of mitochondrial numbers in both groups. Scale bar = 500 nm. J–M) Optical mapping of hEHTs in the PA and control groups at 72 h, stimulated at a pacing frequency of 1 Hz. The results included isochronal activation and propagation direction maps alongside calcium transient traces (J), as well as statistical analysis of Ca^2^⁺ fluorescence signal conduction velocity (K), amplitude (L), and the frequency distribution of two different types of diastolic dysfunction occurrences (M). n = 8 for each group. * *p* < 0.05, ** *p* < 0.01, *** *p* < 0.001, **** *p* < 0.0001.

Given the established presence of severe systolic dysfunction and pronounced cardiac symptoms in diabetic hearts, we investigated whether chronic PA exposure (for 72 h) induces apparent structural damage and impaired systolic function in hEHTs. Our findings revealed a significant decrease in the active force of hEHT in the PA group compared to the control (**Figure** **3**A,B), indicative of reduced systolic function. Concurrently, a marked increase in passive force was observed (Figure 3C), suggesting augmented myocardial stiffness. Furthermore, the PA‐treated group exhibited significantly reduced contraction and relaxation rates (Figure 3D,E). Prolonged contraction and relaxation times, increased APD50/APD80 (Figure S5A–D, Supporting Information), and an elevated proportion of abnormal diastolic peaks were also evident (Figure 3F). Collectively, these results demonstrated impaired systolic and diastolic function in hEHTs after 72 h of PA stimulation.

Furthermore, prolonged PA stimulation (72 h) resulted in a significant increase in LDH release (Figure 3G), indicative of severe hEHT injury. Immunofluorescence staining revealed a substantial increase in fibroblasts within the hEHT, predominantly in the inner region with concomitant cardiomyocyte loss (Figure 3H). Additionally, TEM demonstrated sarcomere disarray and a reduction in mitochondrial number (Figure 3I). Beyond structural damage, 72 h PA exposure led to a marked impairment of calcium signaling propagation in hEHTs, as evidenced by significantly decreased electrical conduction velocity, amplitude, and related parameters with optical mapping method (Figure 3J–L, Figure S5E–G, Supporting Information). Moreover, the incidence of abnormal contraction peaks, particularly twin peaks, was dramatically increased (Figure 3M). Collectively, these findings demonstrate that prolonged PA stimulation recapitulates both systolic and diastolic dysfunction, as well as the structural hallmarks of diabetic cardiomyopathy in the hEHT model.

### 2.4 Short‐Term and Long‐Term PA Exposure Differentially Alters DbCM‐Associated Gene Expression in hEHTs

**Figure 4:**
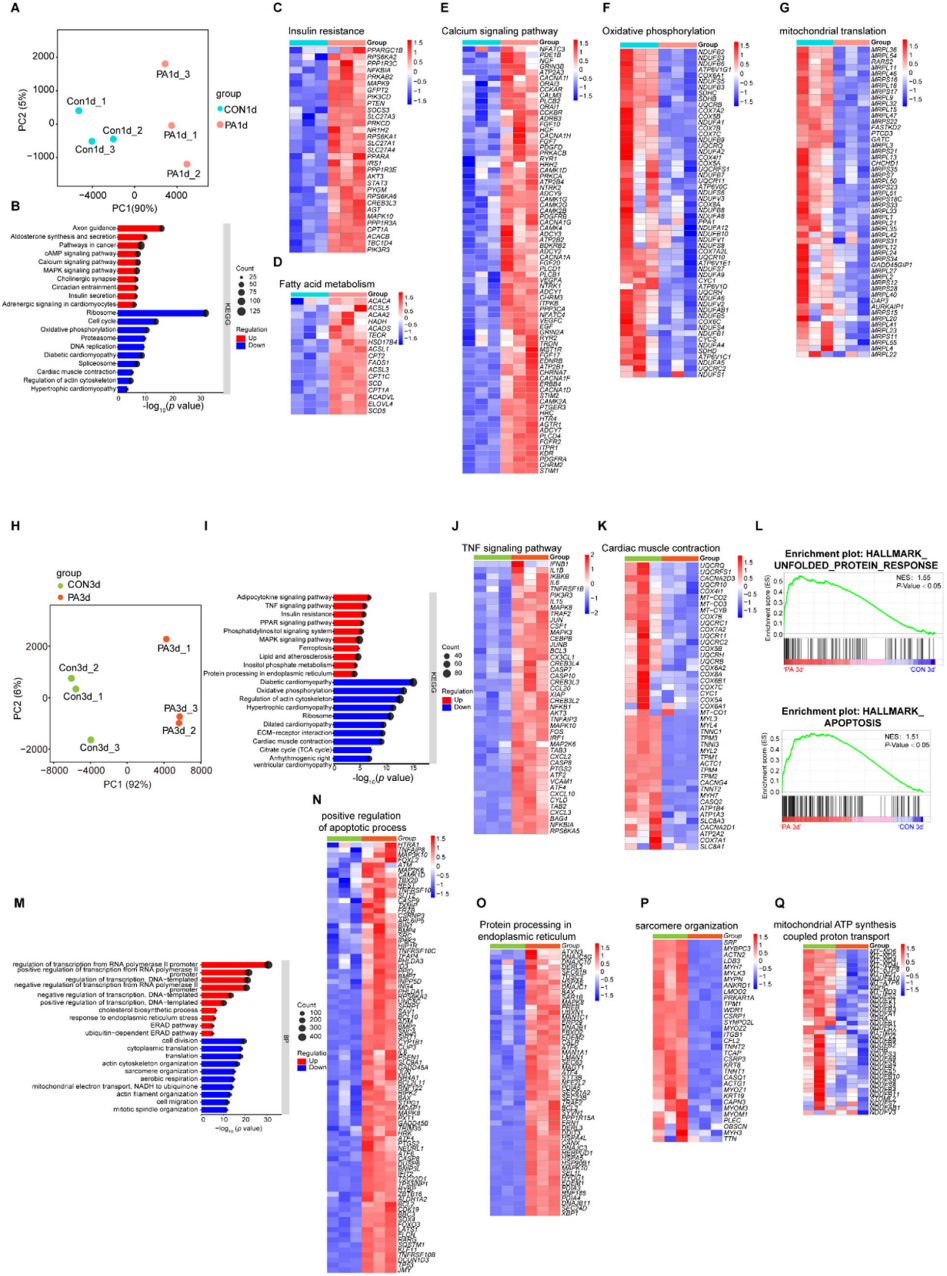
Signaling pathways related to DCM in PA‐induced hEHT models. A) Principal component analysis of the global gene expression profiles between CON1d and PA1d as revealed by RNA‐seq. B–G) Kyoto Encyclopedia of Genes and Genomes (KEGG) pathway enrichment analysis was performed on up‐regulated genes and down‐regulated genes (*p*‐value < 0.05), and the top 10 KEGG pathways were listed with their *p*‐values and counts (B). Heatmaps showing the upregulation of pathways related to insulin resistance (C), fatty acid metabolism (D), and calcium signaling (E), as well as the downregulation of pathways related to oxidative phosphorylation (F) and mitochondrial translation (G) in the PA1d group compared to the CON1d group. H) Principal component analysis of global gene expression profiles between CON3d and PA3d. I–K) KEGG pathway enrichment analysis showed the top 10 pathways with upregulated and downregulated genes, along with their *p*‐values and counts (I). Heatmaps indicated that the TNF signaling pathway (J) was upregulated, while cardiac muscle contraction (K) was downregulated in the PA3d group. L) GSEA showed enrichment of the unfolded protein response and apoptosis pathways in the PA3d group. M) The top 10 biological process terms identified through Gene Ontology enrichment analysis were listed. N–P) Heatmaps displayed the DEGs in BP terms, including upregulation of apoptotic process (N), protein processing in endoplasmic reticulum (O), and downregulation of sarcomere organization (P), mitochondrial ATP synthesis coupled proton transport (Q) in the PA3d group compared with CON3d.

To elucidate the molecular mechanisms underlying short‐term and long‐term PA‐induced injury in hEHTs, we performed RNA‐sequencing analysis in each group at 24 and 72 h. Principal component analysis (PCA) demonstrated a clear separation between PA 24 h and control 24 h hEHT samples (**Figure** **4**A), indicating substantial transcriptomic divergence. KEGG pathway analysis revealed significant downregulation of contractility‐related pathways, including cardiac muscle contraction and oxidative phosphorylation, coupled with upregulation of insulin resistance, fatty acid metabolism, and calcium signaling pathways in the PA group (Figure 4B–F). Notably, gene ontology (GO) analysis of biological processes corroborated the downregulation of mitochondrial translation (Figure 4G), supporting the notion of mitochondrial dysfunction during DbCM processing.

Following 72 h of exposure, principal component analysis (PCA) effectively distinguished the PA group from the control group (Figure 4H). KEGG pathway analysis revealed significant upregulation of pathways associated with protein processing in the endoplasmic reticulum (ER), TNF signaling, and insulin resistance. Conversely, pathways related to cardiac muscle contraction and diabetic cardiomyopathy were significantly downregulated (Figure 4I–K). Consistently, GSEA of these genes revealed enrichment in unfolded protein response and apoptotic pathways (Figure 4L). Furthermore, GO analysis of biological processes identified upregulated genes involved in apoptosis and ER stress pathway (Figure 4M–O), while mitochondrial ATP synthesis coupled to proton transport and sarcomere organization were downregulated in the PA group (Figure 4P,Q). These findings collectively demonstrate that prolonged PA exposure predominantly elicits cell apoptosis and ER stress, contributing to subsequent structural and functional impairments. Given the critical roles of mitochondrial dysfunction and ER stress in diabetic cardiomyopathy,^[^ ^8^ ^]^ our model exhibits a high degree of functional and molecular similarity to this condition, suggesting its utility as a valuable in vitro platform for further investigations.

### 2.5 Empagliflozin Ameliorates PA‐Induced Diastolic Dysfunction in iPSC‐CM and hEHT Models

**Figure 5:**
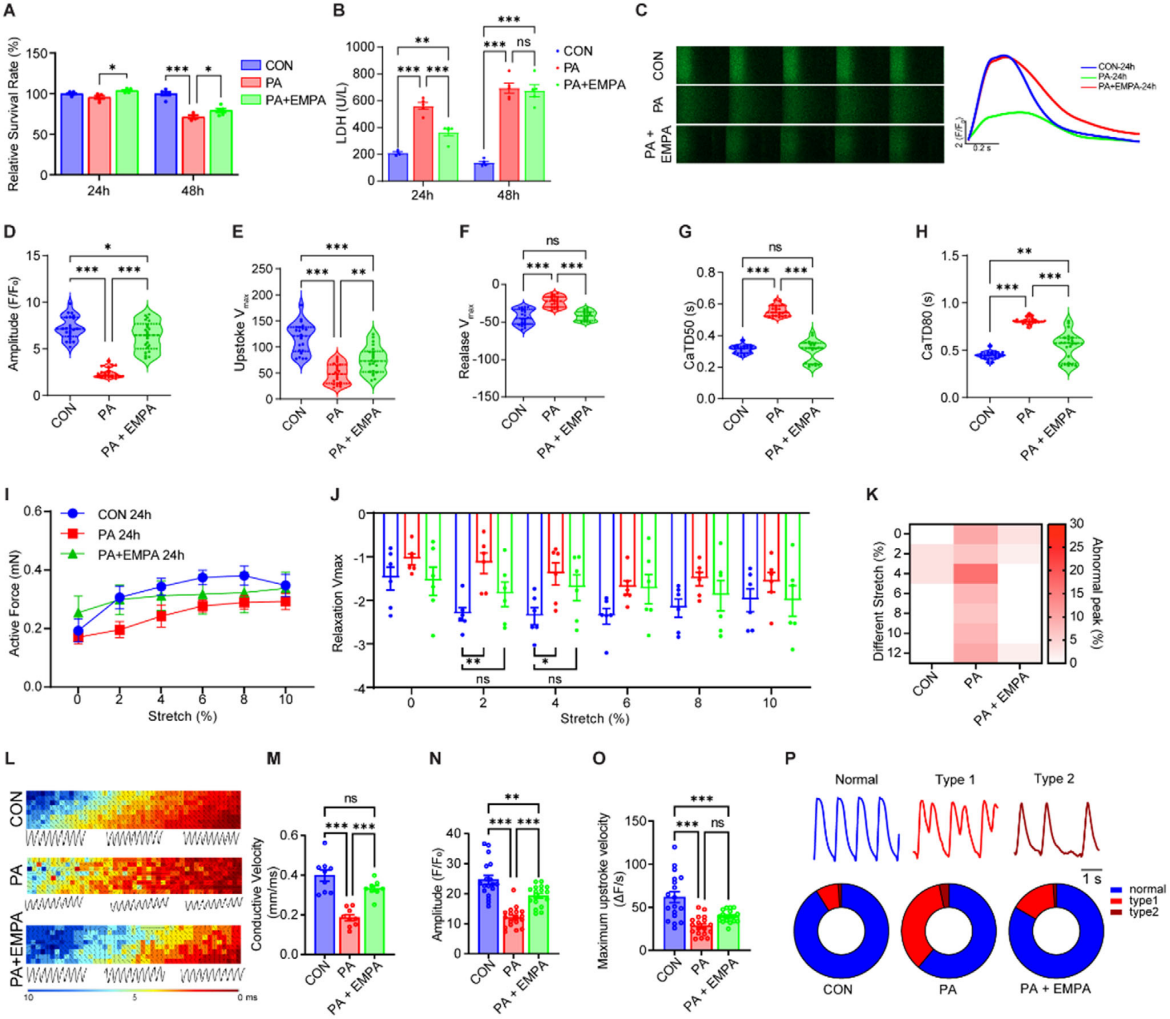
Protective Role of Empagliflozin against PA‐Induced Injury in iPSC‐CMs and hEHTs. A,B) CCK8 (A, n = 5) and LDH (B, n = 5) assays of iPSC‐CM treated‐with PA, PA + empagliflozin (EMPA) and control for 24 h and 48 h. C–H) Representative images (C) and quantification of Ca^2+^ transient in iPSC‐CMs from PA, PA+EMPA and control group after 24 h. iPSC‐CMs were loaded with Fluo‐4 AM and paced at 1 Hz, the parameters including amplitude (D), maximum upstroke velocity (E), maximum release velocity (F), the duration at 50% and 80% repolarization (CaTD50/CaTD80) (G,H) were analyzed. I,K) Statistical analysis of contraction force, including active contractile force (I), maximum relaxation velocity (J), and heatmaps showing the percentage of diastolic dysfunction peaks (K) after 24 h of treatment in PA, PA+EMPA and control group. n = 6 per group. L–P) Optical mapping of hEHTs in the PA, PA+EMPA and control groups at 24 h, stimulated at a pacing frequency of 1 Hz. The results included isochronal activation and propagation direction maps alongside calcium transient traces (L), as well as statistical analysis of Ca^2^⁺ fluorescence signal conduction velocity (M), amplitude (N), maximum upstroke velocity (O), and the frequency distribution of two different types of diastolic dysfunction occurrences (P). n = 9 for the CON group, n = 9 for the PA group, n = 8 for the PA+EMPA group. ns *p* > 0.05, * *p* < 0.05, ** *p* < 0.01, *** *p* < 0.001.

To investigate the potential therapeutic efficacy of empagliflozin, a frontline medication for diabetes, in the PA‐induced lipotoxic DbCM model, we initially assessed the effects of EMPA on cardiomyocyte viability using CCK‐8 assay. Our findings demonstrated that EMPA significantly improved cardiomyocyte viability at both 24‐ and 48‐h post‐PA exposure (**Figure** **5**A), although LDH levels were only reduced at the 24‐h timepoint (Figure 5B). Furthermore, empagliflozin restored calcium handling capacity, including calcium transients, release rate, reuptake rate, as well as CaTD50 and CaTD 80, in iPSC‐CMs exposed to PA for 24 h (Figure 5C–H). Noting that SGLT2 inhibitors typically achieve relatively low serum concentrations, far below 22 µM (≈10 mg),^[^ ^59^, ^60^ ^]^ we conducted a dose‐response analysis which revealed that only high concentrations of empagliflozin (EMPA) significantly ameliorated PA‐induced cardiomyocyte injury (Figure S6A–D, Supporting Information). These results suggest that high‐dose empagliflozin exhibits early cardioprotective effects in this PA‐induced cardiomyopathy model.

To further investigate the potential protective effects of empagliflozin in early‐stage diabetic cardiomyopathy, we focused on hEHTs exposed to short‐term PA stimulation. Empagliflozin significantly ameliorated early‐stage diastolic dysfunction in hEHTs, as evidenced by slight improvements in active and passive force, significantly recovered contraction and relaxation rates, and APD50 (Figure 5I,J, Figure S7A–C, Supporting Information). Notably, empagliflozin reduced the incidence of abnormal diastolic peaks compared to the PA group (Figure 5K). Furthermore, empagliflozin treatment attenuated PA‐induced impairments in electrical conduction velocity and amplitude, and markedly decreased the occurrence of abnormal contraction peaks (Figure 5L–P). These findings suggest that empagliflozin may mitigate lipotoxic myocardial injury, particularly during the early phases of diastolic dysfunction.

**Figure 6:**
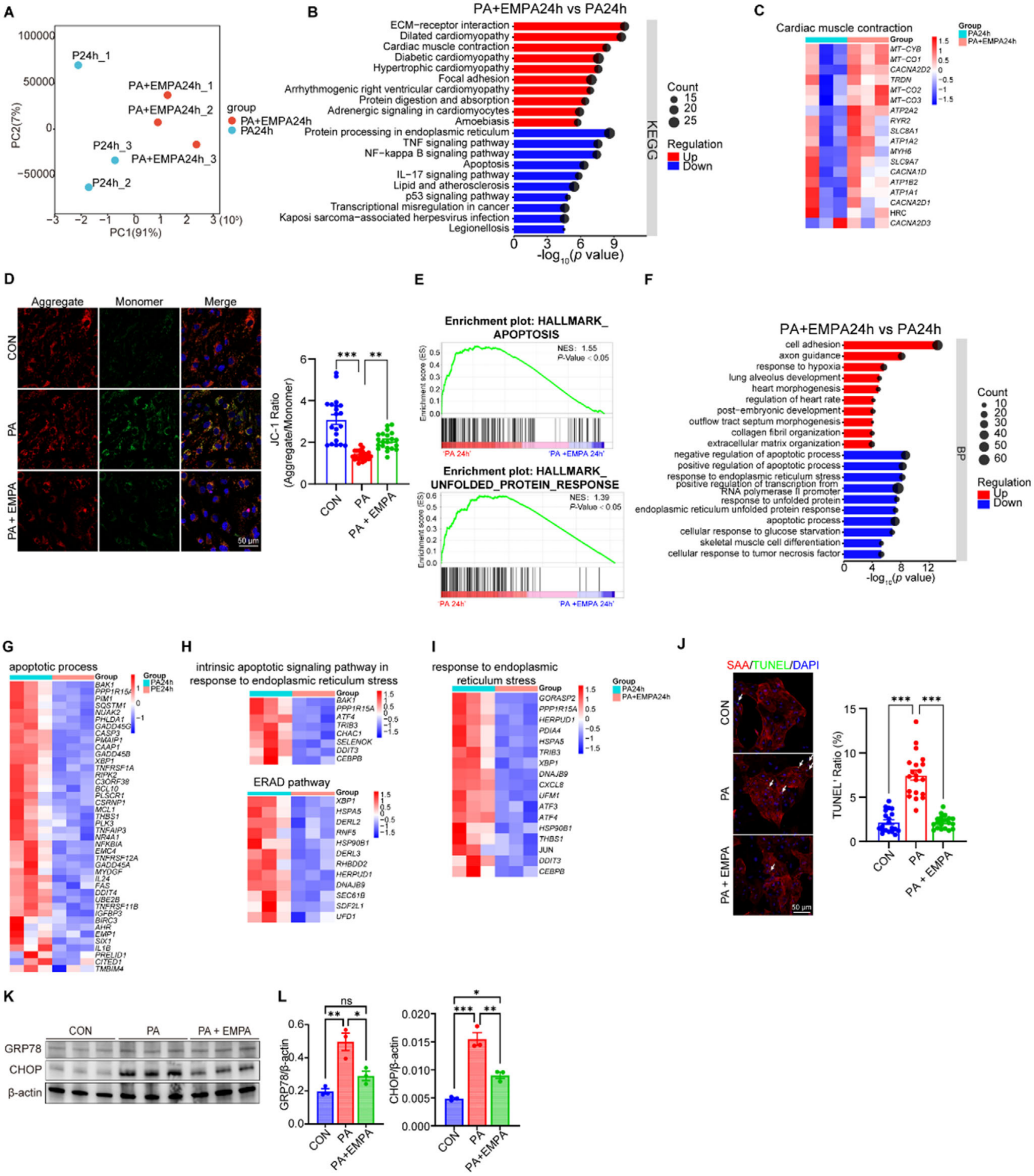
Empagliflozin Protects hEHT and iPSC‐Derived Cardiomyocytes from Palmitate‐Induced Injury by Inhibiting Mitochondrial Dysfunction, Apoptosis, and ER Stress. A) Principal component analysis of global gene expression profiles between PA24h and PA+EMPA24h. B,C) KEGG pathway enrichment analysis showed the top 10 pathways with upregulated and downregulated genes, along with their *p*‐values and counts (B). Heatmaps showing the upregulation of pathways related to cardiac muscle contraction in the PA+EMPA group compared to the PA group (C). D) Representative fluorescent image (left) and quantification (right) of mitochondrial membrane potential measure by JC‐1 staining in iPSC‐CMs. Red fluorescence indicates the mitochondrial JC‐1 aggregate JC‐1, while green fluorescence represents the monomeric form of JC‐1. Bars = 50 µm. E) GSEA showed enrichment of apoptosis and unfolded protein response pathways in the PA group. F) The top 10 biological process terms identified through Gene Ontology enrichment analysis were listed. G–I) Heatmaps displayed the DEGs in BP terms, including downregulation of apoptotic process (G) and ER stress (H‐I) in the PA+EMPA group compared with the PA group. J) Representative fluorescent images (left) and quantification (right) of apoptotic cardiomyocyte rates in iPSC‐CMs were assessed by TUNEL assay in the CON, PA, and PA+EMPA groups at 24 h. Bars = 50 µm. K‐L) Immunoblotting analyses of GRP78 and CHOP protein levels in hEHTs treated with CON, PA, or PA+EMPA for 24 h. β‐Actin was used as a loading control. n = 3 biological replicates. ns *p* > 0.05, *p < 0.05, **p < 0.01, ***p < 0.001.

To elucidate the mechanisms underlying empagliflozin's protective effects against PA‐induced diastolic dysfunction in hEHTs, RNA‐seq analysis was performed (**Figure** **6**A). KEGG pathway analysis revealed that empagliflozin treatment upregulated pathways associated with cardiomyopathy, primarily involving genes related to cardiac muscle contraction, especially those involved in mitochondrial transcription and ATP synthesis (Figure 6B,C). Thus, JC‐1 staining was performed and revealed that EMPA ameliorates the PA‐induced decrease in mitochondrial membrane potential in iPSC‐CMs (Figure 6D). Meanwhile, both KEGG and GSEA analyses indicated downregulation of apoptosis, ER stress, and inflammatory pathways in the EMPA+PA group (Figure 6B,E, Figure S8A–D, Supporting Information). Moreover, GO analysis identified a reduction in apoptotis and ER stress, ERAD, and unfolded protein response‐related genes in the PA + empagliflozin group (Figure 6F–I). To validate these sequencing results, additional experiments were conducted. The anti‐apoptotic effect of EMPA was confirmed by TUNEL staining in iPSC‐CMs (Figure 6J). Protein expression analysis of key pathway components revealed that ER stress‐related proteins were significantly upregulated by PA treatment but markedly reduced by EMPA after 1 day in hEHTs (Figure 6K,L). Collectively, these results suggest that empagliflozin ameliorates PA‐induced hEHT injury by suppressing apoptosis and ER stress, while concurrently promoting cardiac structure and function.

To investigate why EMPA exerts a direct protective effect against PA‐induced injury in hiPSC‐CMs and hEHTs despite the negligible expression of SGLT2, we analyzed the reported potential targets of SGLT2 inhibitors in our hEHT sequencing data. We found that the FPKM value of SGLT2 was extremely low, whereas its homolog SGLT1 was readily detectable, a finding further confirmed by western blotting (Figure S9A,B,C Supporting Information). Additionally, other potential targets including Nav1.5, NHE1, and NCX1 were also exhibited relatively high FPKM values (Figure S9A, Supporting Information). Importantly, protein levels of SGLT1 and NHE1 did not show significant changes after treating hEHTs with PA or PA+EMPA for 1 day (Figure S9B,C Supporting Information), suggesting that EMPA likely inhibits channel activity rather than regulating protein expression under these conditions.

### 2.6 Empagliflozin Protects Mice from HFD + STZ Induced Functional Impairment and Electrical Uncoupling

**Figure 7:**
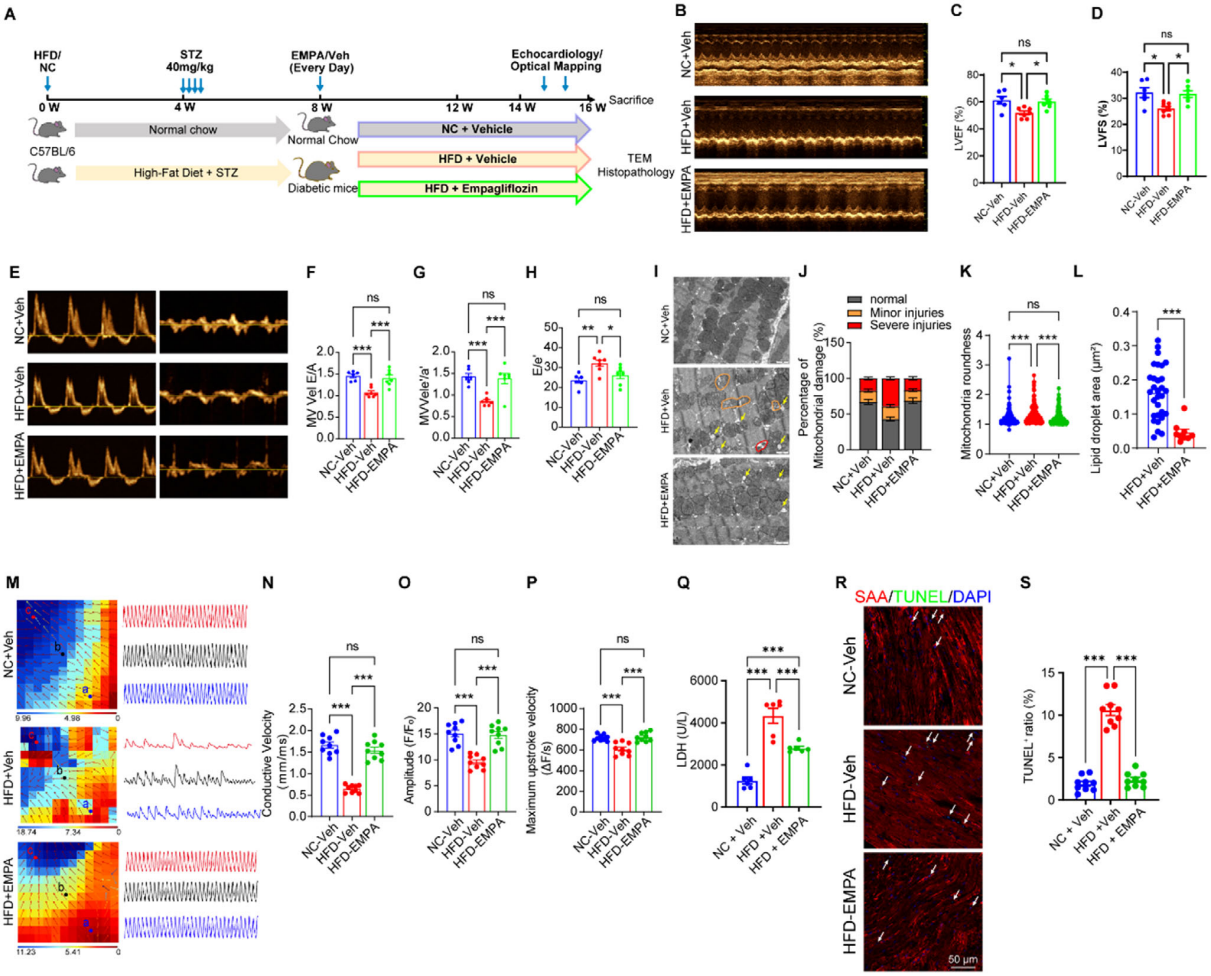
Empagliflozin protects against cardiac injury in T2DM mice. A) Schematic protocol of T2DM induction and EMPA administration in C57/BL6 mice. B–H) Representative images of echocardiography (B, E) and quantification of parameters including left ventricular ejection fraction (LVEF, C) and left ventricular Fractional shortening (LVFS, D), Mitral Valve Velocity E/A Ratio (F), e“/a” Ratio (G) and E/a' Ratio (H) from the NC + vehicle group (n = 6), the HFD + Vehicle group (n = 7), and the HFD + Empagliflozin group (n = 7). I,J) Representative TEM images (I) and quantitative analysis of mitochondrial roundedness (J) from each group. Yellow arrows indicated lipid droplets, orange circles indicated mild mitochondrial damage, and the red circles represented severe mitochondrial damage. K) The area of lipid droplets was quantified in the HFD+EMPA and HFD+veh groups. (n = 10 images per group). M–P) Optical mapping of hearts isolated from the NC+veh, HFD+Vehicle, and HFD+EMPA group stimulated at 8 Hz pacing. Statistical analysis of Ca^2^⁺ fluorescence signal conduction velocity (N), amplitude (O), and maximum upstroke speed (P) were derived from over three recordings from one mouse per group. Q) Lactate dehydrogenase (LDH) levels were measured in the serum of mice from the NC‐Veh, HFD‐Veh, and HFD+EMPA groups (n = 6 mice per group). R,S) Representative fluorescent images (R) and quantification (S) of apoptotic cardiomyocyte rates in heart sections from each group are shown. Data were obtained from n = 3 mice per group, with at least three high‐power fields analyzed per mouse. Bars = 50 µm. ns *p* > 0.05, *p < 0.05, **p < 0.01, ***p < 0.001, ****p < 0.0001.

To further validate the protective role of empagliflozin (EMPA) in a type 2 diabetes mellitus (T2DM) model, we employed a murine model induced by a high‐fat diet in conjunction with low‐dose streptozotocin (STZ) injections (**Figure** **7**A). Hyperglycemic mice were subsequently treated with either daily gavage of EMPA or a saline control for 8 weeks. Echocardiographic analysis revealed that T2DM mice exhibited systolic dysfunction, characterized by a decrease in ejection fraction and fractional shortening (Figure 7B–D). Doppler echocardiography indicated elevated E/e' ratios and decreased E/A and e“/a” ratios in T2DM mice compared to controls, suggesting the presence of diastolic dysfunction (Figure 7E–H). Notably, EMPA treatment significantly attenuated these functional impairments (both systolic and diastolic) and structural dysfunctions (Figure 7B–H). Ultrastructural analysis demonstrated significant mitochondrial injury and lipid droplet accumulation in the hearts of T2DM mice, which were markedly improved following EMPA treatment (Figure 7I–L). Furthermore, ventricular electrical conduction, assessed via activation mapping, revealed uniform electrical conduction with orderly propagation in control hearts. In contrast, T2DM hearts displayed disordered left ventricular conduction with aberrant conduction sites. The T2DM+EMPA group exhibited restored conduction patterns, correlating with improved conduction velocity, amplitude, and Vmax velocity (Figure 7M–P). Last, EMPA reduced the LDH content in the serum of T2DM mice heart (Figure 7Q), and the anti‐apoptotic effect of EMPA was further confirmed by TUNEL staining of mouse paraffin‐embedded heart sections (Figure 7R,S). Collectively, these results demonstrated that EMPA protects against diabetes‐induced cardiac dysfunction and abnormalities in electrical conduction.

## 3 Discussions

Diabetes poses a significant public health threat due to its substantial contribution to cardiovascular disease (CVD) morbidity and mortality, particularly heart failure.^[^ ^3^, ^4^, ^5^ ^]^ However, the underlying mechanisms of diabetic myocardial disorder/diabetic cardiomyopathy remain elusive due to the complex interplay of risk factors and systemic effects. Traditional animal models often replicate the end‐stage events observed in clinical patients, making it challenging to distinguish primary from secondary factors and to establish causal relationships between risk factors and eventual outcomes. Therefore, the development of in vitro tissue models that accurately recapitulate these processes is essential for advancing effective prevention and treatment strategies for heart failure in the T2DM population. iPSCs‐related models have emerged as a robust substitute for animal testing in preclinical drug development, as validated by the FDA.^[^ ^61^ ^]^ However, an ideal DbCM model that can fully elucidate the complex interplay of risk factors leading to heart failure is still lacking. In this study, our hEHT model recapitulated the detrimental effects of excess saturated fatty acids under controlled high glucose culture conditions, leading to impaired staged myocardial diastolic and systolic function, coupled with all‐stagey abnormal electrical conduction, as well as myocardial structural damage, mitochondrial dysfunction, and cardiac fibrosis. Notably, our results first demonstrated that empagliflozin directly attenuated HFD/PA induced functional decrease and electrical abnormality both in mouse model and hEHT model, and reduced PA‐induced endoplasmic reticulum stress, calcium dysregulation, and apoptosis in hEHT, which are early‐stage contributors to diastolic dysfunction. These findings provide compelling evidence supporting the clinical application of empagliflozin for heart failure with preserved ejection fraction (HFpEF)‐related myocardial symptoms.

Although the exact definition and clinical features of diabetic cardiomyopathy or diabetic myocardial disorder remain controversial, there is an undeniable association between T2DM and the development of HF, and it emphasizes that even patients with T2DM who have asymptomatic cardiac abnormalities are at increased risk for HF.^[^ ^62^ ^]^ Notably, the detection of asymptomatic structural and functional cardiac abnormalities through imaging modalities, often manifested as early diastolic dysfunction,^[^ ^14^, ^15^ ^]^ is crucial for identifying individuals at high risk of HF. Previous study utilized murine myocardium‐derived EHTs treated with AGEs recapitulated the phenomenon of increased ROS, inflammatory pathway, cardiac hypertrophy and fibrosis by western blot and qPCR.^[^ ^36^ ^]^ Another study used heart organoid with pregestational diabetes‐like conditions to revealed the alteration of epicardial and cardiomyocyte dysfunction and lipid metabolism.^[^ ^37^ ^]^ However, these models lacked tissue‐level functional change as the new definition emphasized systolic and/or diastolic dysfunction. Our system supports the investigation of contraction force and electrophysiological function dynamically, which allows more choices for understanding the contributions of different diabetic risk factors for further study.

Mitochondrial dysfunction, ER stress, and inflammation are key factors in the development and progression of diabetic cardiomyopathy.^[^ ^9^ ^]^ Notably, human diabetic hearts exhibit an increased reliance on fatty acid‐based energy production^[^ ^63^ ^]^ yet show reduced ATP efficacy and heightened mitochondrial fragmentation.^[^ ^64^, ^65^, ^66^ ^]^ Our hEHT model effectively recapitulated these characteristics, as evidenced by mRNA sequencing that revealed a downregulation of genes involved in mitochondrial translation (Figure 4G). Concurrently, there was an upregulation of genes associated with the ER unfolded protein response (UPR), TNF signaling, and insulin resistance pathways (Figure 4C,J,O). Transmission electron microscopy (TEM) analysis demonstrated changes in mitochondrial morphology, which aligned with elevated expression of fatty acid oxidation genes at 24 h (Figure 4D). However, this was accompanied by a decline in contractile function and electrical conduction capacity observed at 72 h (Figure 3A–M), indicating a dynamic transition from initial mitochondrial function impairment to subsequent dysfunction.

In this study, we developed an in vitro tissue model to investigate lipotoxic myocardial injury. Our findings suggest that EMPA directly ameliorates abnormal diastolic peaks and Ca^2^⁺ activity resulting from palmitate acid‐induced dysfunction. This protective effect is primarily mediated through the inhibition of apoptosis, mitochondrial injury, and ER stress, highlighting EMPA's direct impact on cardiac function. As a newly FDA‐approved combination treatment for heart failure in individuals with or without T2DM, EMPA's cardioprotective mechanisms remain incompletely understood, warranting further investigation. Although SGLT2 is virtually undetectable in the heart, its homolog SGLT1 is the predominant SGLT family member expressed in the myocardium and can be inhibited by SGLT2 inhibitors.^[^ ^67^, ^68^ ^]^ SGLT1 is recognized as an important potential target in cardiovascular disease.^[^ ^68^, ^69^, ^70^, ^71^, ^72^ ^]^ Overexpression of SGLT1 in mice leads to pathological hypertrophy and cardiac dysfunction, while knockdown attenuates these effects.^[^ ^73^ ^]^ Moreover, SGLT1 plays a significant role in glucose fluctuation‐induced cardiac injury via oxidative stress and mitochondrial dysfunction.^[^ ^74^ ^]^ In addition, several studies have demonstrated that SGLT2is^[^ ^51^ ^]^ possess potential inhibitory activity against various sodium‐related channels, including sodium‐hydrogen antiporter (NHE1),^[^ ^75^, ^76^, ^77^, ^78^ ^]^ Nav1.5,^[^ ^52^, ^79^, ^80^ ^]^ and NCX1.^[^ ^81^, ^82^ ^]^ We analyzed the expression of these potential targets in our sequencing data and found that SGLT2 expression (FPKM) was nearly zero, while other genes were detectable (Figure S9A, Supporting Information). Western blotting confirmed the protein levels of SGLT1 and NHE1, with no significant changes observed at either the transcriptional or translational level when hEHTs were treated with PA+EMPA compared to PA alone for 1 day (Figure S9B–C, Supporting Information). Although EMPA exhibits over 2000‐fold higher selectivity for SGLT2 (IC_50_: 3.1 nM) than for SGLT1 (IC_50_: 8.3 µM),^[^ ^83^ ^]^ our dose‐response assays revealed that only high concentrations of EMPA provided significant protection against PA‐induced cardiomyocyte injury (Figure S6A–D, Supporting Information), preserved mitochondrial membrane potential (Figure 6D), and improved hEHT function (Figures 4, 5, 6). These results suggest that SGLT1 may be a key target through which EMPA exerts its protective effects in our system. However, we cannot exclude the involvement of other sodium‐related channels. For instance, NCX1 has been reported to participate in ER stress in neurons and islet β‐cells,^[^ ^84^, ^85^, ^86^ ^]^ truncated variants of Nav1.5 can initiate the unfolded protein response (UPR) in the ER in systolic human heart failure,^[^ ^87^ ^]^ and NHE1 transgenic mice exhibit an increased ER stress response in the myocardium.^[^ ^88^ ^]^ In the future, the generation of SGLT1 and other candidate gene knockout iPSC‐derived cardiomyocytes and hEHTs could be utilized to specifically identify the primary targets of EMPA's protective mechanisms in this context.

Our results support the notion that EMPA enhances both systolic and diastolic function, as well as electrophysiological function, in a diet‐ and drug‐induced DbCM model. This finding is consistent with observations from other animal models, such as db/db mice, where EMPA has been shown to alleviate oxidative stress and mitochondrial dysfunction,^[^ ^89^ ^]^ coronary microvascular dysfunction,^[^ ^90^ ^]^ and improving diastolic function.^[^ ^91^ ^]^ Moreover, EMPA has been reported to improve endothelial function by reducing mitochondrial reactive oxygen species (ROS) in endothelial cells (ECs), which helps decrease frailty in diabetic and hypertensive patients.^[^ ^92^ ^]^ Our results offer additional insights into the beneficial actions of EMPA in injured tissue models. However, the precise targets of EMPA and SGLT2 inhibitors within the myocardium, along with their underlying mechanisms, still require further clarification through both clinical evidence from heart samples and further experimental studies. This remains a critical area for future research.

In summary, we have developed an engineered DbCM model that simulates lipotoxic myocardial injury, characterized by early‐stage diastolic impairment and late‐stage systolic dysfunction. Utilizing this model, we provide direct evidence that EMPA improves PA‐induced hEHT injury by inhibiting mitochondrial injury, ER stress, and cell death, which suggests that EMPA may help alleviate diabetic diastolic dysfunction.

## 4 Experimental Section

### hiPSC Culture and Cardiomyocyte Differentiation

Human iPSC line WTC (Gladstone Institute, derived from fibroblast) was used. Cardiac differentiation was conducted following the Wnt signaling protocol as previously described.^[^ ^42^ ^]^ Briefly, hiPSCs were dissociated to single cells (Versene, Thermo Fisher, 15 040 066) and reseeded onto Matrigel‐coated (Corning, 354 262) plates at a density of 3.0 × 10^4^ cells cm^−2^. Cells were cultured in mTeSR1 medium (Stemcell, 85 850) supplemented with Y‐27632 (Stem cell, 72 308) and incubated at 37 °C with 5% CO_2_ for 24 h. Then, the medium was replaced with a complete mTeSR1 medium without Y‐27632 and changed every 24 h. When the confluence reached 80%, the medium was switched to differentiation basal medium (RPMI 1640 containing B27‐insulin and 200 µg mL^−1^ ascorbic acid (Sigma A8960)) and treated with 7.5 µM CHIR99021 (Tocris, 4423). After 48 h, the medium was replaced with differentiation basal medium for 24 h. Next, 5 µM IWR‐1 (Tocris, 3532) was added to the differentiation basal medium for 48 h. IWR‐1 was then removed, and differentiation basal medium was changed every 48 h until day 14 of differentiation. From day 14 onward, the cardiomyocytes were used either for generation of 3D hEHTs or reseeded onto plates for further assays.

### Flow Cytometry

Cardiomyocytes derived from hiPSCs were dissociated into single cells using 0.25% trypsin‐EDTA at room temperature for 5 min. Harvested cells were then washed twice with DPBS (containing 2% FBS) in a 15 mL centrifuge tube and fixed with 4% paraformaldehyde (Biosharp, BL539A) for 15 min. Following three washes with DPBS, the cells were blocked with blocking buffer (DPBS containing 5% donkey serum and 0.2% Triton X‐100) at 4 °C overnight. Next, the cells were incubated with PE‐CTNT (1:20, BD Pharmingen, 564 767) in blocking buffer at 4 °C for 1 h. After washing twice with DPBS, the cells were resuspended in 50–120 µL of DPBS for flow cytometry analysis.

### CCK8 Assay

Seed 14‐d differentiated iPSC‐CMs at a density of 0.75 million cells well^−1^ in a 96‐well plate containing 200 µL of culture medium per well. Cells were cultivated for 3 days before starting the experiment. In the experimental groups with different factors (PA, Glu, and AGE), or in the PA experimental group, cells were exposed to different concentrations (200, 500, and 750 µmol L^−1^) of Palmitic acid (PA, Sigma, P5585) and incubated for 24 or 72 h. Then, 10 µL well^−1^ of CCK 8 reagent (Yeasen,40203ES76) was incubated with 90 µL well^−1^ of cell culture medium mixture for 2 h, and the absorbance value (A) was measured at a wavelength of 450 nm using a Paradigm Molecular Devices (Gemini XPS). Cell viability was calculated as [(As‐Ab)/(Ac‐Ab)] ×100%, where “As” is the absorbance value of experimental well, “Ac” is the absorbance value of control well, and Ab is the absorbance value of blank well.

### Lactate Dehydrogenase Release Assay

Cell cytotoxicity was measured using an LDH assay kit (Nanjing Jiancheng Bioengineering Institute; A020‐2‐2) according to the manufacturer's protocols. Briefly, 20 µL of supernatant samples were collected from cardiomyocytes stimulated with different stimulies and loaded onto a 96‐well plate. Set up control and experimental groups, each with five replicates. Prepare the reaction system according to the experimental protocol (no coenzyme I in the control group, and addition of coenzyme I in the experimental group). Mix well and incubate at 37 °C for 15 min, followed by the addition of 2,4‐dinitrophenylhydrazine and further incubation at 37 °C for 15 min. The mixture was then incubated with NaOH solution room temperature for 5 min. The absorbance value (A) of each well was measure at a wavelength of 450 nm.

### Mitochondrial and Endoplasmic Reticulum Morphology Analysis

HiPSC‐CMs were seeded on confocal dishes and treated with PA at different time points. To visualize mitochondrial or endoplasmic reticulum morphology changes, cardiomyocytes were incubated in a working solution containing 1 µM Mitotracker Red (Thermo Fisher, M22425) or ER Tracker Green (Thermo Fisher, E34251) at 37 °C with 5% CO_2_ for 15 min. Subsequently, Hoechst staining was applied for 10 min, followed by washing twice. After that, the cells were incubated with 2.5 µM Blebbistatin (Sigma, B0560) for 3 min to suppress cardiomyocyte contractions. Imaging was performed using an LSM 980 inverted laser scanning confocal microscope with a 63× oil immersion lens. Data analysis was conducted using the MINA plugin in Fiji‐ImageJ software.

### Ca^2+^ Transient and Intracellular Calcium Content Measurement

HiPSC‐CMs were incubated with a working solution of 2.5 µM Fluo‐4 AM (Thermofisher, F14201) for 20 min at 37 °C in a 5% CO_2_ humidified incubator. The staining solution was then aspirated, and the cardiomyocytes were then incubated in a working solution of 2.5 µM Blebbistatin for 3 min to inhibit contractions. Calcium transients were recorded using line scan mode of LSM 980 microscopy at 1 Hz with a 63× oil immersion objective. Calcium activity changes within the CMs were recorded for 10 s. To assess intracellular calcium content, cardiomyocytes were placed in calcium‐free Tyrode's solution (140 mM NaCl, 5 mM KCl, 2 mM MgCl_2_, 10 mM glucose and 10 mM HEPES at pH 7.4). Throughout the recordings, the cells were maintained in a heated chamber at 37 °C. Calcium transient data were analyzed using custom PyCharm and MATLAB programs.

### Measurement of Mitochondrial Membrane Potential

Mitochondrial membrane potential (MMP) was assessed using the JC‐1 dye (Thermo Fisher, T3168) following the manufacturer's instructions. Briefly, hiPSC‐CMs were washed and incubated with JC‐1 dye (1:1000 dilution) and 1 mg mL^−1^ Hoechst at 37 °C for 20 min, then washed again before imaging with a laser confocal microscope. The red fluorescence emitted by JC‐1 aggregates was detected at an emission wavelength of 572 nm, while the green fluorescence from JC‐1 monomers was monitored at 517 nm. The ratio of red (aggregate) to green (monomer) fluorescence was quantified using ImageJ software to evaluate changes in mitochondrial membrane potential. A decreased JC‐1 ratio indicates mitochondrial depolarization, reflecting a loss of mitochondrial membrane potential.

### hiPSC‐Derived 3D hEHTs Construction and Culture

The hEHT was constructed as previously described.^[^ ^40^ ^]^ On day 14 of differentiation, 1.2 × 10^6^ cells were added into 58 µL 1× cardiac media consisting of RPMI 1640 medium (Gibco, C11875500BT), 2 µg mL^−1^ Vitamins B12 (Sigma‐Aldrich, V2876), 2 mg mL^−1^ 6‐Aminocaproic acid (Sigma‐Aldrich, A2504), 1% Penicillin‐Streptomycin (Thermo Fisher, 15 140 122), 10% FBS and 2.4 µL 50 U mL^−1^ thrombin (Sigma‐Aldrich, T7201). Then the cardiac medium was mixed with the hydrogel solution consisting of 24 µL 2× cardiac medium, 24 µL 10 mg mL^−1^ fibrinogen (Sigma‐Aldrich, F4883), and 12 µL Matrigel (Corning, 354 277), transferred to the PDMS model and incubated at 37 °C for 30 min to form two bundle hEHTs. The hEHTs were subsequently placed in cardiac culture medium (containing differentiation basal medium with 1 mM sodium pyruvate (Gibco, 11360070), 2 mg mL^−1^ 6‐Aminocaproic acid (Sigma, A2504), 0.1 mM non‐essential amino acids (Gibco, 11140050), 0.45 mM 1‐Thioglycerol (Sigma, M6145) and 2% FBS) and cultured on a rocker. During the initial 24 h, 10 µM Y‐27632 and 5% FBS were supplemented to the cardiac culture medium to optimize the viability of iPSC‐CMs. After removing 10 µM Y‐27632 and 5% FBS, the cardiac culture medium was changed every 48 h until day 9, when stimulations began. Palmitic acid (Sigma, P5585) and BSA (Yeasen, B9301110) were used as control treatments starting on the ninth day until harvest.

### hEHT Contraction Force Measurement

After treatment with 500 µM PA alone or in combination with 22 µM Empagliflozin (HY‐15409; MedChemExpress) for the indicated time points, hEHTs were placed in the customized force‐testing apparatus described previously.^[^ ^40^ ^]^ Briefly, hEHTs were subjected to electrical field stimulation at voltages of 10 V and different frequencies (0, 0.5 Hz, 1 Hz, 1.5 Hz, 2 Hz, 2.5 Hz) in a bath solution containing 1.8 mM CaCl_2_. Subsequently, the tissue strips were incrementally stretched to 12% of their original length at a rate of 2% per increment under conditions of 10 V and 1.5 Hz. Each stretch level was recorded for 30 s. Contraction force data were analyzed using custom MATLAB programs.

### Animal Experiments

8‐week‐old male C57BL/6J mice were purchased from Henan Skbes Biotechnology Co., Ltd. (Henan, China) and were maintained under a 12‐h light/dark cycle. All procedures were performed according to the NIH guidelines. The study protocols and use of animals were approved by the Hubei University Animal Ethics and Welfare Committee (20220012). Mice in the control group were allowed ad libitum access to water and a regular chow diet, with 14.5% fat, 27.38% protein, and 58.12% carbohydrate (cat. no.1035, BEIJING HFK BIOSCIENCE CO.LT, China). High‐fat diet (HFD) with 60 kcal% fat, 20 kcal% carbohydrate, 20 kcal% protein, was purchased from Research Diets, Inc. (D12492, USA).

Mice were acclimated to the environment for 1 week and then randomly assigned to either the normal chow group (NC) or the HFD group. After 4 weeks, the HFD‐fed mice were fasted overnight and received intraperitoneal injections of streptozotocin (50 mg kg^−1^, S0130, Sigma) for four consecutive days. Mice with blood glucose levels ≥11.1 mmol L^−1^ were selected and further divided into the empagliflozin group and the vehicle group. Empagliflozin was dissolved in DMSO (10 mg mL^−1^) and administered to T2DM mice at a dosage of 10 mg kg^−1^ via oral gavage daily. Mice in NC group and the HFD‐vehicle group received an equal volume of saline and DMSO by gavage. After 8 weeks, cardiac function was evaluated using echocardiography. Subsequently, the animals were sacrificed, and their hearts were excised for further analyses. n = 6 mice in NC + vehicle group, n = 7 in HFD + Vehicle group, and n = 7 mice in HFD + Empagliflozin group.

### Echocardiography

Cardiac echocardiography was performed on mice anesthetized with 1–1.5% isoflurane using the VINNO V6 Vet system, the heart rate was maintained between 400 and 450 beats per minute to facilitate Doppler studies. Images were acquired with a X10‐23L transducer. Fractional shortening (FS), left ventricular ejection fraction (LVEF), ventricular dimensions, and volumes were assessed via a M‐mode. Passive left ventricular filling peak velocity (*E*, mm s^−1^) and atrial contraction flow peak velocity (*A*, mm s^−1^) were obtained by mitral valve Doppler flow. Early (*e*’, mm s^−1^) and late (*a*′, mm s^−1^) diastolic mitral annular motion velocity was obtained by tissue Doppler imaging from the apical 4 chamber view.

### Transmission Electron Microscopy

The EHT‐bundle and mice heart were directly fixed with a sufficient amount of pre‐cooling 2.5% glutaraldehyde and transferred to 1.5 mL EP tubes for fixation and stored in the refrigerator at 4 °C. This process should be gentle and quick to make sure not to damage cells or tissues. The hEHT samples were sent to Wuhan University People's Hospital for transmission electron microscopy preparation and imaging. The heart samples were sent to Wuhan Luochuang Biotechnology Co., Ltd. for subsequent preparation and imaging. Data analysis was performed using Fiji‐ImageJ software.

### Optical Mapping in hEHT and Mice

After 7 days of culture, hEHTs underwent optical mapping of transmembrane potentials using voltage‐sensitive fluorescent dyes and a high‐speed camera. hEHT were co‐incubated with Rhod‐2 AM (Thermo Fisher, R1245MP, 2.5 µM) at 37 °C for 25 min. Following two washes with DPBS, hEHT were cultured in tissue culture medium supplemented with 2.5 µM Blebbistatin (Sigma, B0560) to suppress cardiomyocyte contraction and eliminate motion artifacts during recording. Electrical activity induced by stimulation with point electrodes was recorded for 10 s using a 594‐channel photodiode array in microscopy mode (16×). Conduction velocity (CV) data analysis was performed using BV Ana software. Membrane potential amplitude, APD50, APD80, maximum upstroke velocity, and maximum decay rate were analyzed using the PyCharm program. Rainbow mapping was performed for analysis using OMapScope.

Similar to the methods described before,^[^ ^42^ ^]^ the mice were anesthetized and euthanized to remove the heart. The heart was placed on the Langendorff perfusion apparatus (Scope, SG‐LP011) and the aorta was retrogradely perfused with K‐H solution containing 119 mM NaCl, 4 mM KCl, 1.8 mM CaCl_2_‐2H_2_O, 1 mM MgCl_2_‐6H_2_O, 1.2 mM KH_2_PO_4_, 25 mM NaHCO_3_ and 10 mM glucose at 37 °C and 10 mL min^−1^ flow rate. After 15 min of stabilization, 1 mg mL^−1^ blebbistatin (Abcam, ab120425) was perfused into the heart to eliminate motion artifacts. Intracellular calcium changes were measured using 1 mg mL^−1^ RHOD‐2AM (Abcam, ab142780). The entire heart was illuminated with 549 nm excitation light (MappingLab, LEDC‐2001), and CMOS cameras (MappingLab, OMS‐PCIE‐2002) were used to capture the fluorescent signal emitted by the heart. The intracellular calcium signal under 8 Hz stimulation (stimulation at the apex of the heart with twice the threshold current) was recorded. The commercially available analysis software OMapScope 4.0 (MappingLab, Version 5.7.8) and BV‐Ana Analyzer (BrainVision, Version 16.04.20) were applied for processing the optical mapping data from miceearts and hEHTs. Gaussian spatial filtering (3 × 3 pixels) and median filtering (3 × 3 pixels) were used for the spatial alignment and processing of the calcium signal.

### Histological Analysis

hEHTs were fixed with 4% paraformaldehyde at room temperature for 20 min, then dehydrated, embedded in paraffin, and sectioned into 6‐µm‐thick slices. Subsequently, tissue sections were deparaffinized and rehydrated for further staining. For immunostaining of tissue fibrosis, antigen retrieval was first performed using pH 6.0 citric acid solution, followed by blocking with blocking solution at 37 °C for 1 h. Next, hEHT sections were incubated with rabbit anti‐ Vimentin (CST #5741, 1:1000 dilution) and goat anti‐cTnI (Thermo Fisher Scientific, # PA1‐86820, 1:1000 dilution) primary antibodies at 4 °C overnight. After washing away excess primary antibodies with DPBS, sections were incubated with secondary antibodies for 2 h, followed by counterstaining of cell nuclei with DAPI (Invitrogen, 62 248, 1:1000). The level of fibrosis was analyzed using Image J software to quantify and analyze the percentage of Vimentin positive area.

The harvested mice hearts were fixed in 4% paraformaldehyde for 24–36 h at 4 °C, halved 1–2 mm above the ligature knot, dehydrated in an ethanol and xylene series, and embedded in paraffin. hearts were serially sectioned at 6 µm thickness from the heart, H&E was performed using standard procedures.

### TUNEL Staining

Apoptosis was detected using the In Situ Death Detection Kit (YEASEN, 40306ES50). For cellular‐level analysis, iPSC‐derived cardiomyocytes (iPSC‐CMs) were cultured in confocal dishes. Cells were fixed with 4% paraformaldehyde (PFA) at room temperature for 15 min in the dark, followed by permeabilization and blocking with a solution containing 0.2‰ Triton X‐100 and 5% donkey serum at 37 °C for 30 min. Samples were then incubated overnight at 4 °C with the SAA primary antibody (Sigma‐Aldrich, A7811). After washing with PBS, TUNEL staining was performed according to the manufacturer's instructions, followed by incubation with the appropriate fluorescent secondary antibody.

For TUNEL staining of mouse heart tissue, 6 µm‐thick paraffin sections were first deparaffinized, and antigen retrieval was conducted using citrate buffer (pH 6.0). The subsequent staining procedure was identical to that used for cellular apoptosis detection. Finally, images were acquired using a confocal fluorescence microscope (Zeiss, LSM 980). The apoptosis rate was expressed as the proportion of apoptotic cardiomyocytes relative to the total number of cells.

### Western Blot Analysis

Briefly, hEHT samples from three experimental groups (CON, PA, and PA + Empagliflozin), each with three biological replicates, were lysed using RIPA buffer (Beyotime, China) supplemented with protease and phosphatase inhibitors (Roche, Switzerland). Protein concentrations were determined using the BCA protein assay kit (Thermo Fisher Scientific, USA). Equal amounts of protein (15–20 µg) were separated on 4–20% gradient FuturePAGE precast gels (15‐well format, ET15420Gel; ACE Biotechnology, ChangZhou, China) and transferred onto PVDF membranes (Bio‐Rad, USA). Membranes were blocked with 5% non‐fat milk in TBST buffer (Tris‐buffered saline with 0.1% Tween‐20) for 2 h at room temperature, then incubated overnight at 4 °C with the following primary antibodies: GRP78 (ABclonal, A23453, Rb, 1:6000); CHOP (ABclonal, A21902, Rb, 1:1000); SGLT1 (ABclonal, A26241, Rb, 1:1000); NHE1 (Proteintech, 67363‐1‐ig, Ms, 1:5000); β‐actin (Servicebio, GB11001, Ms, 1:1000). After washing with TBST, membranes were incubated for 2 h at room temperature with HRP‐conjugated secondary antibodies against rabbit (Proteintech, SA00001‐2, 1:5000) or mouse (ABclonal, AS003, 1:5000). Following three washes, protein bands were visualized using ECL reagent (Immobilon Western Chemiluminescent HRP Substrate, WBKLS0100) and detected with a chemiluminescence imaging system (Bio‐Rad, USA). Results were expressed as density values normalized to β‐actin levels.

### RNA Sequencing and Gene Expression Analysis

The differential gene expression analysis of RNA‐seq data was conducted using the Npvaseq PE150 platform from GeekGene (Beijing, China) based on Illumina Novaseq technology with paired‐end sequencing. briefly, hEHTs were stimulated with PA, empagliflozin, or BSA control for 1 or 3 days before harvesting using TRIzol (ThermoFisher Scientific, 15596026) according to the manufacturer's instructions. Each group included of 3 biological replicates. For downstream analysis, FPKM lists were used. Principal component analysis (PCA) was conducted on the GeekGene platform (http://www.geekgene.com.cn/). Gene Ontology (GO) and Kyoto Encyclopedia of Genes and Genomes (KEGG) pathway enrichment analyses were performed by submitting gene lists to the DAVID bioinformatics tool., A cutoff of *p*‐value < 0.05 and an absolute fold‐change ≥ 1.5 was used to identify differentially upregulated or downregulated genes. The top 15 most significant gene entries were then analyzed further. Volcano plots and heatmaps were generated to visualize the significantly differentially expressed genes using the bioinformatics platform (https://www.bioinformatics.com.cn/). For Gene Set Enrichment Analysis (GSEA), normalized FPKM lists were analyzed using GSEA_4.3.2 software for downstream analysis.

### Statistical Analysis

The data were examined to assess whether they met the assumptions of the selected statistical tests. Data analysis was performed using the Prism software package (GraphPad Software). Results were expressed as the mean ± standard error (SEM). An unpaired *t* test was used to compare two groups, whereas an ordinary ANOVA with Tukey's correction was used to compare data from more than two groups. Statistical significance was considered when *p* < 0.05.

## Conflict of Interest

The authors declare no conflict of interest.

## Author Contributions

L.C., Y.Z., and Z.L. contributed equally to this study. L.C. and D.Z. conceptualized the study. L.C., Y.Z., Z.L., and D.Z. designed the experiments; Y.Z., Z.L., L.X., and Y.W. conducted experimental studies in hPSC culture, model establishment, contraction force assessment, optical mapping, and sample collection. L.X. and Z.L. performed bulk RNA sequencing analysis. L.C., Z.L., and Y.W. conducted experiments on the T2DM model in mice. S.W. and Z.L. performed optical mapping in mice. Q.L. and Y.Y. provided some of the preliminary experimental results of cells and hEHTs before graduation. L.C., Y.Z., Z.L., L.X., Y.W., S.W., Q.L., Y.Y., and D.Z. analyzed the experimental data and organized the figures. L.C., Y.Z., Z.L., L.X., Y.G., and D.Z. wrote or edited the manuscript.

## Supporting information



Supporting Information

## Data Availability

The datasets used and/or analyzed during the current study are available from the corresponding author on reasonable request.
