# Supplementary material for: A Human Engineered Heart Tissue‐Derived Lipotoxic Diabetic Cardiomyopathy Model Revealed Early Benefits of Empagliflozin
Source: Adv Sci (Weinh). 2025 May 28;12(30):e03173. doi: 10.1002/advs.202503173 (PMC12376570; doi:10.1002/advs.202503173)
Supplement: Supplementary file 1 — Supporting Information [file ADVS-12-e03173-s001.docx]

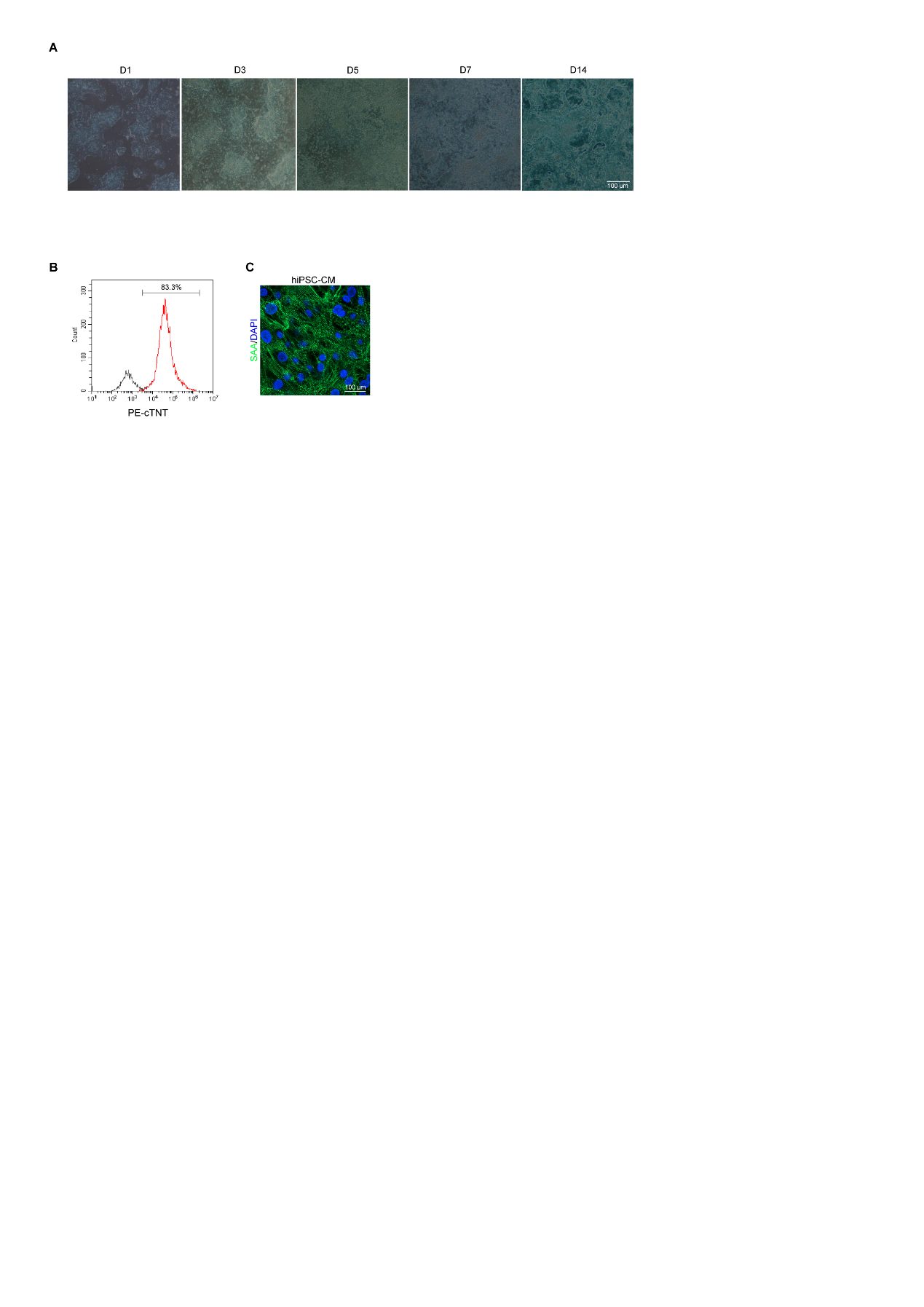


**Supplementary Figure. S1: Differentiation of human iPSC into cardiomyocyte.** (**A**) Representative phase contrast images illustrating changes in cell morphology during iPSC cardiomyocyte differentiation on days 1, 3, 5, 7, and 14. Scale bar = 100 µm. (**B**)  Flow cytometry analysis of the CTNT positive rate at day 14 of iPSC differentiation. (**C**) Representative immunofluorescence staining of cardiomyocyte marker SAA (green) and nucleus (DAPI, blue) of iPSC-CMs.


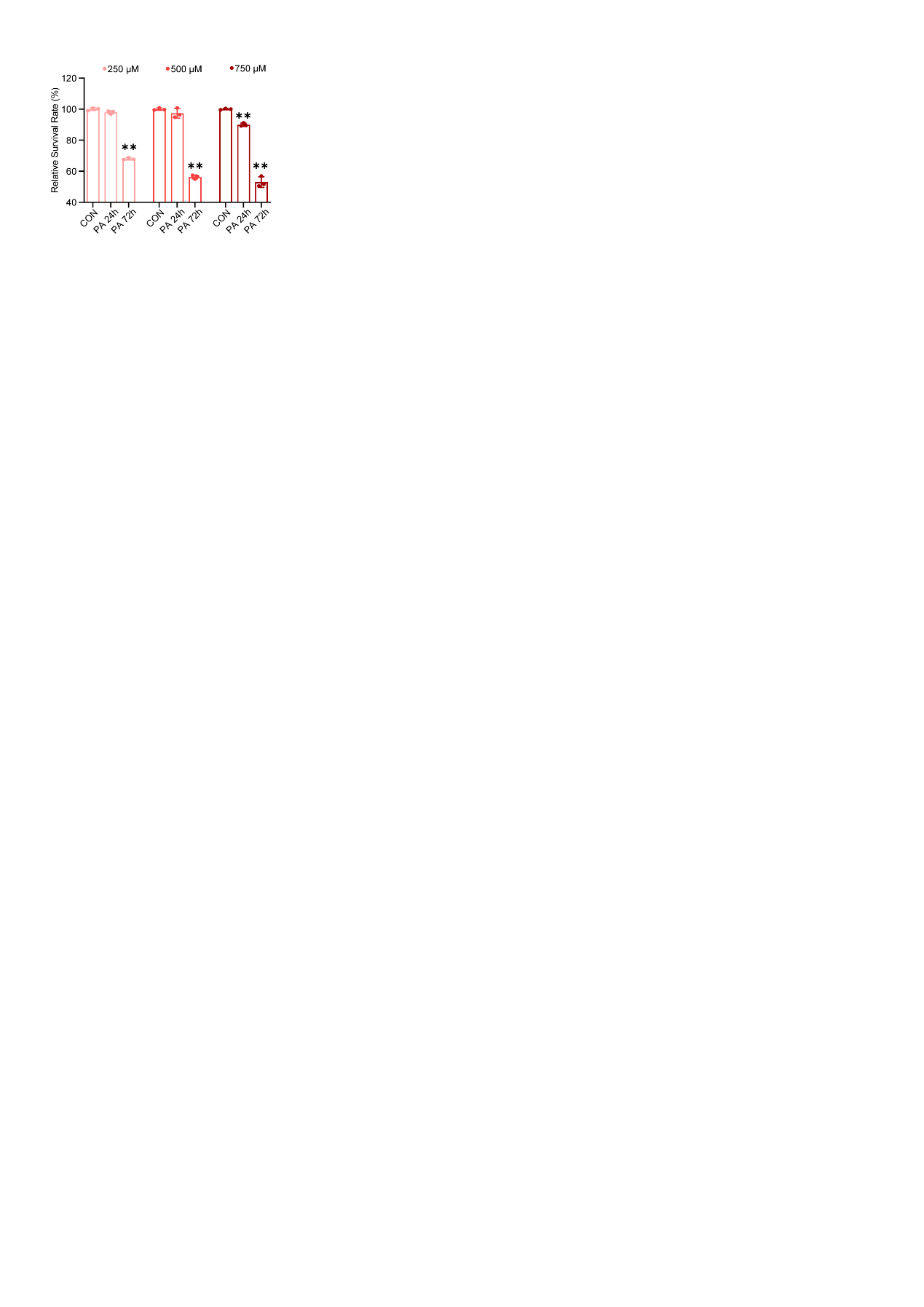


**Supplementary Figure. S2: Relative survival rate of iPSC-CMs following palmitic acid (PA) treatment.** CCK8 assay showing the survival rate of iPSC-CMs treated with PA in a time- and dose-dependent manner. ** *p* < 0.01 vs the indicated CON group.


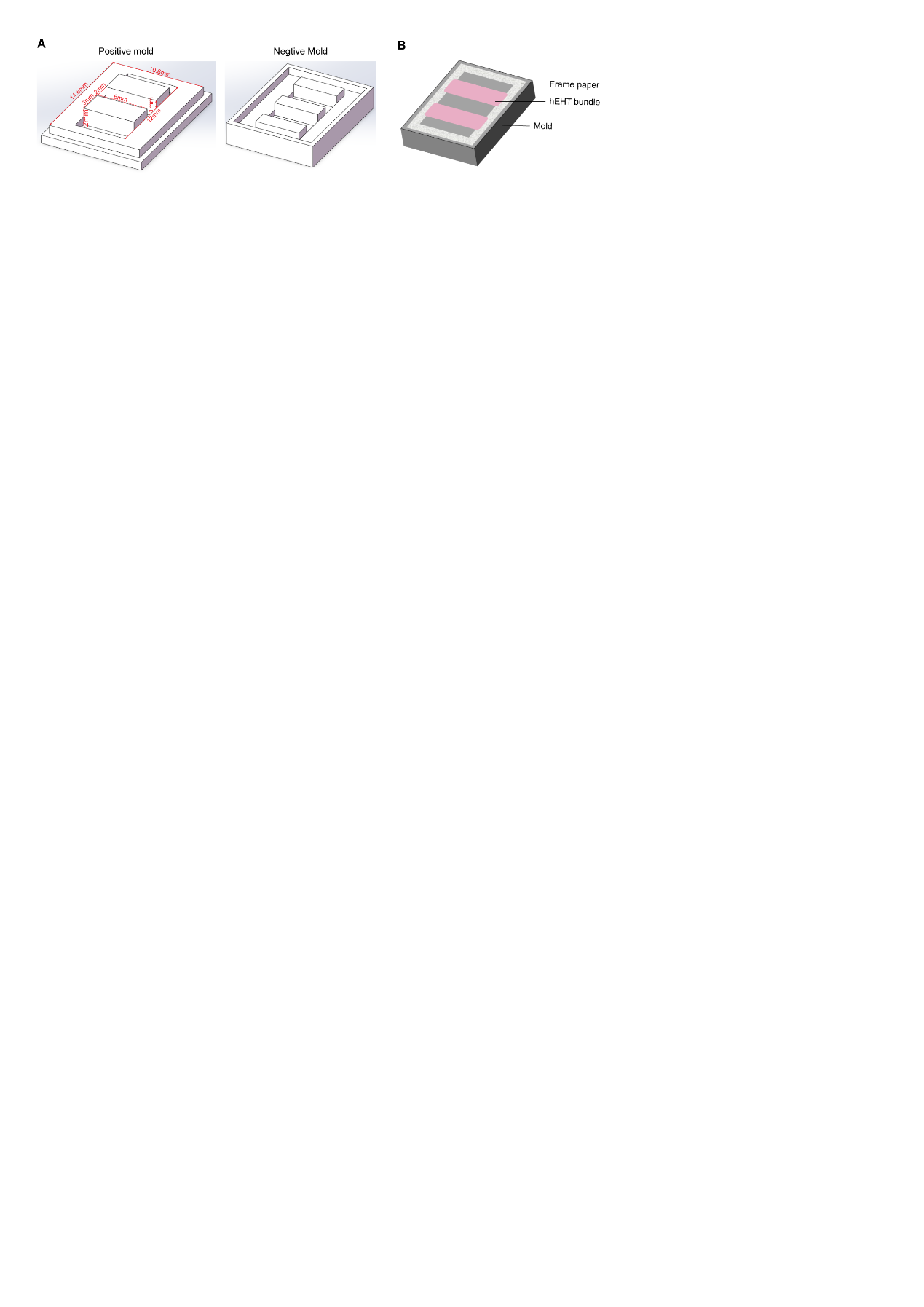


**Supplementary Figure. S3: Schematic diagram of hEHT mold construction.** (**A**) Schematic diagram illustrating the detailed parameters of the positive and negative molds for hEHT. (**B**) Schematic representation of hEHT within the PDMS mold.


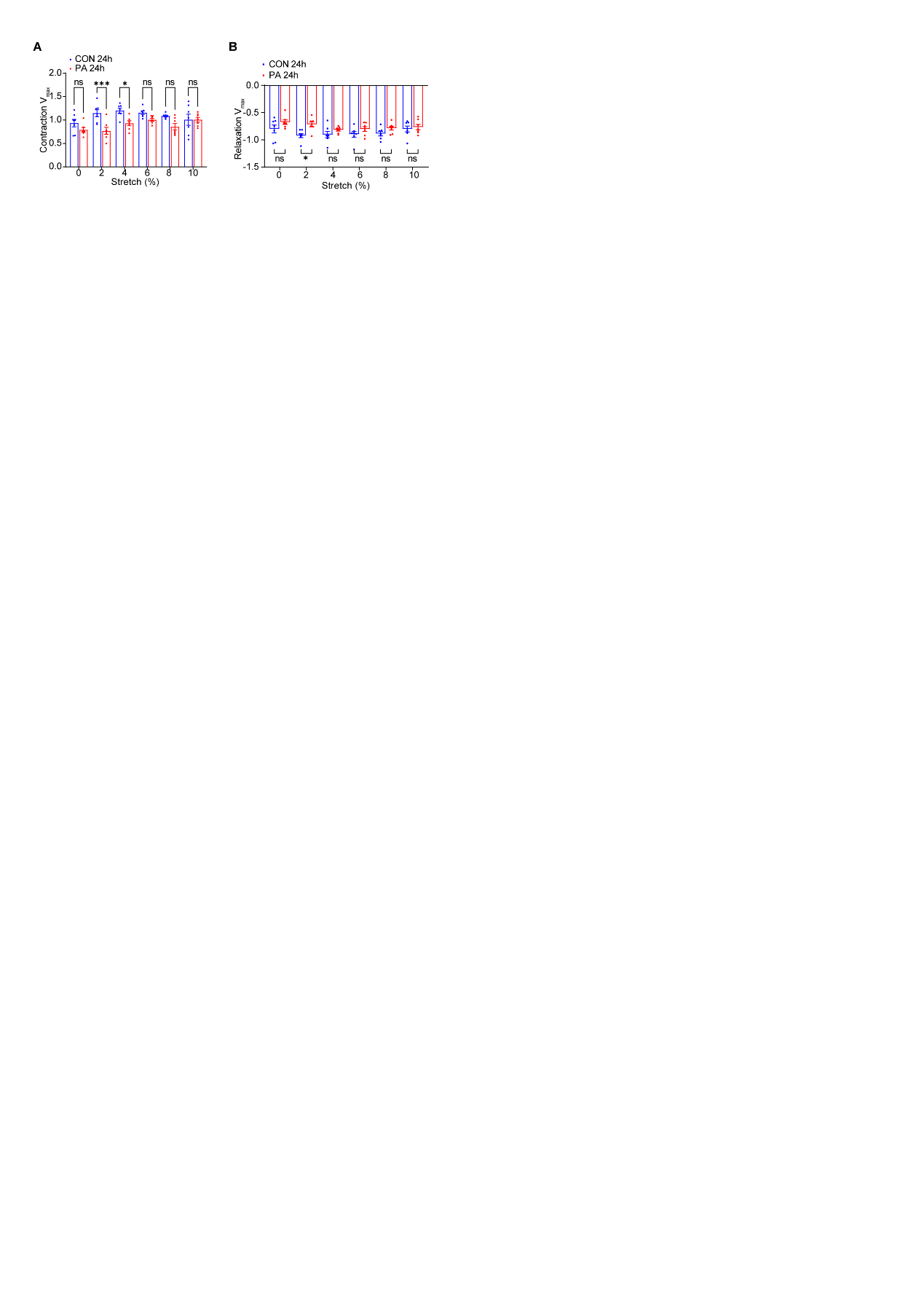


**Supplementary Figure. S4: Parameters of contractile properties between PA-treated hEHT and the control group after 24 hours.**  (**A-B**) Statistical analysis of maximum contraction velocity (**A**) and maximum relaxation velocity (**B**) under different stretching conditions of hEHT during progressive stretching with electrical stimulation (1.5 Hz) after 24 h of treatment in PA stimulation (n = 6 per group). ns *p* ＞ 0.05, * *p* < 0.05, *** *p* < 0.001.


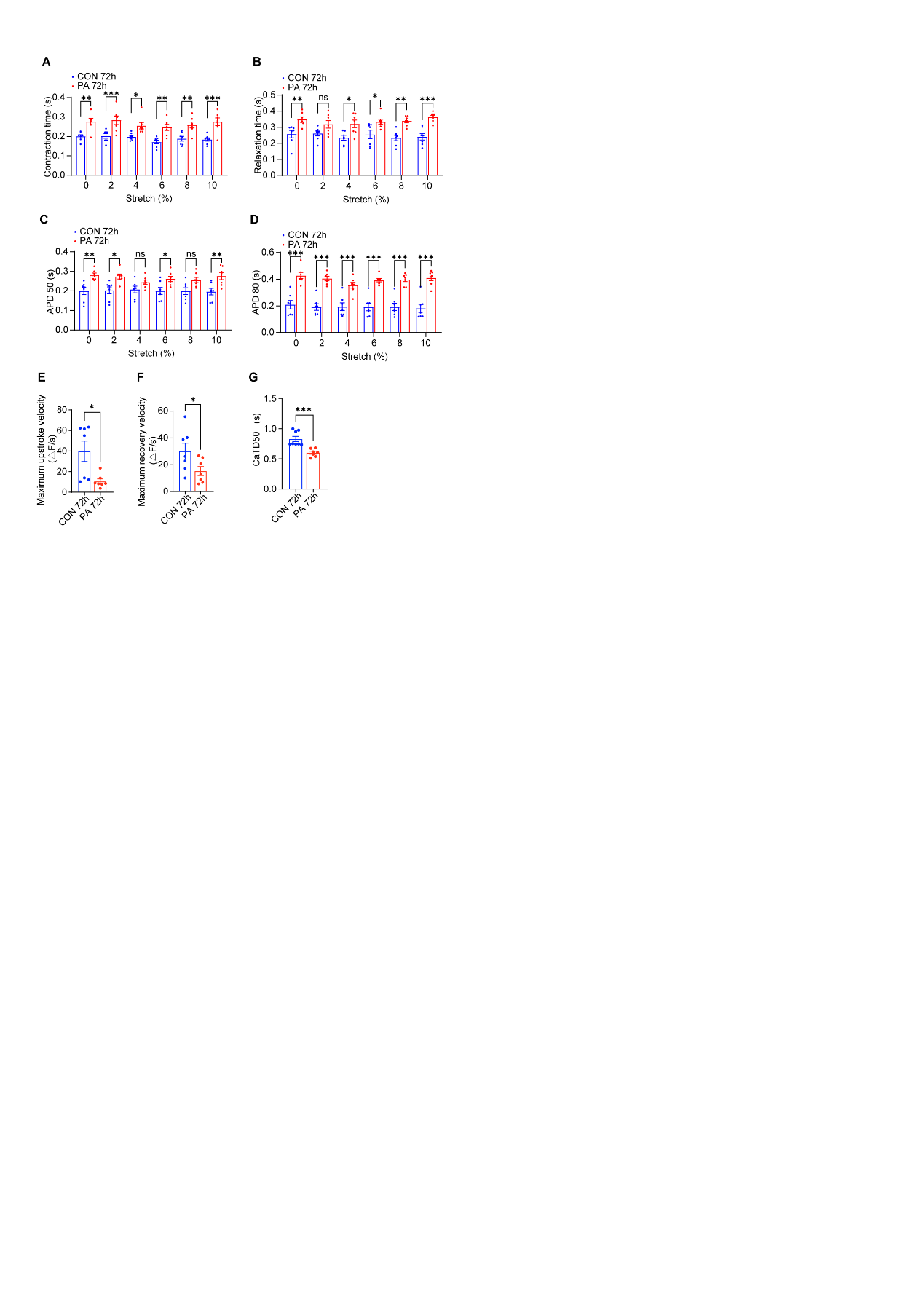


**Supplementary Figure. S5: Parameters of mechanical and electrophysiological functions in hEHTs stimulated by PA for 72 h.** (**A**-**D**) Statistical analysis of parameters including contraction time (**A**), relaxation time (**B**), action potential duration at 50% repolarization (APD 50) (**C**), and action potential duration at 80% repolarization (APD 80) (**D**) under different stretching conditions of hEHT during progressive stretching with electrical stimulation (1.5 Hz) after 72 h of treatment in PA stimulation (n = 7 per group). (**E**-**G**) Optical mapping of hEHTs in the PA and control groups at 72 h, stimulated at a pacing frequency of 1 Hz. The results included maximum upstroke speed (**E**), maximum recovery speed (**F**) the duration at 50% repolarization (CaTD50) (**G**). n = 8 for each group. ns *p* ＞ 0.05, * *p* < 0.05, ** *p* < 0.01, *** *p* < 0.001.


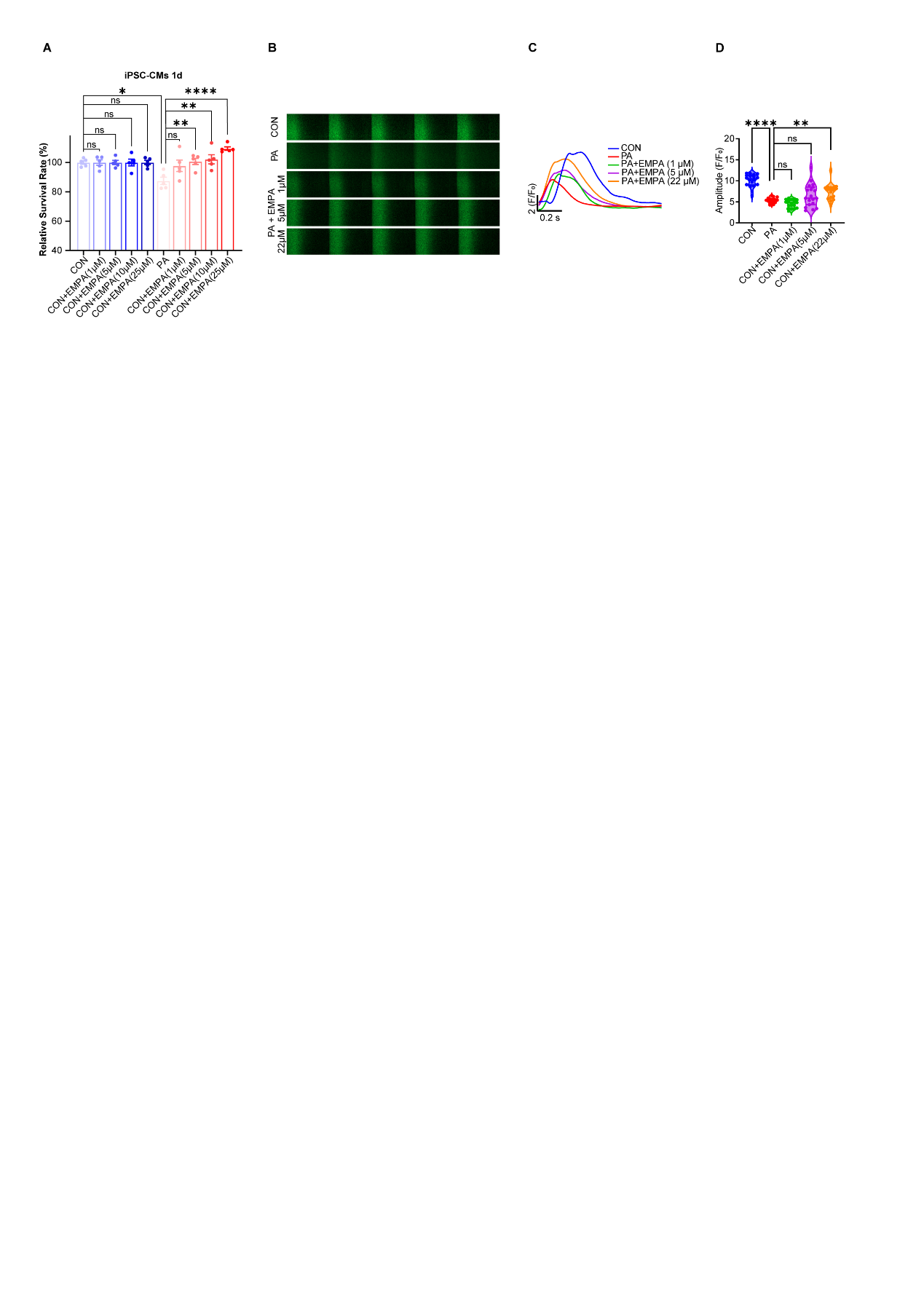


**Supplementary Figure. S6: Dose-dependent effects of EMPA on iPSC-CMs after 1 day of PA treatment.** (**A**) CCK-8 assay showing the effects of various concentrations of EMPA on iPSC-CMs with or without PA treatment. (**B**-**D**) Representative images (**B**), characteristic Ca²⁺ transient peaks (**C**), and amplitude measurements (**D**) of Ca²⁺ transients in iPSC-CMs treated with different concentrations of EMPA under PA administration and control conditions after 24 hours. ns *p* ＞ 0.05, * *p* < 0.05, ** *p* < 0.01, **** *p* < 0.0001.


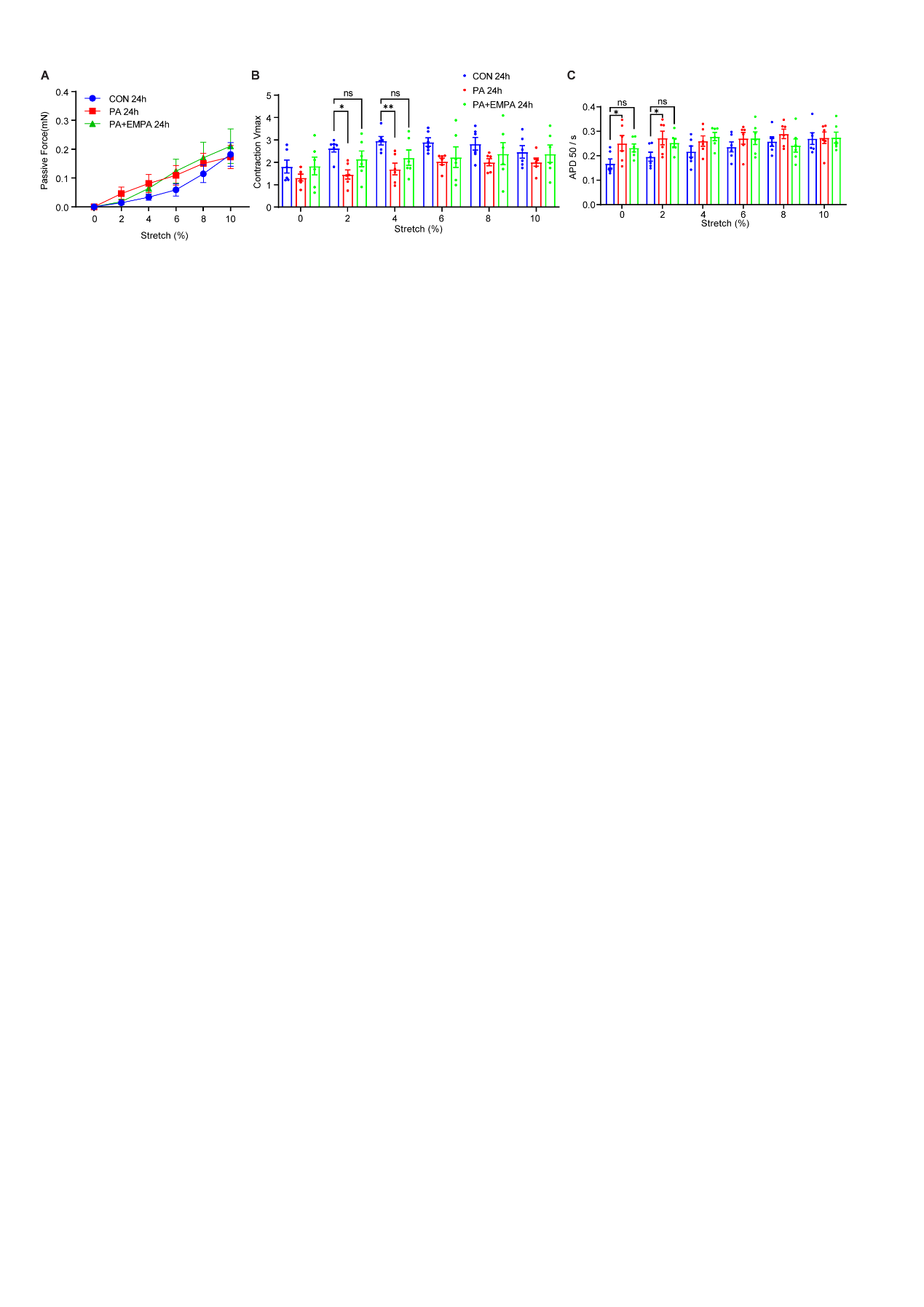


**Supplementary Figure. S7: Parameters of contractile properties among the PA, PA + empagliflozin, and control groups after 24 hours.** (**A-C**) Statistical analysis of passive contraction force (**A**), maximum contraction velocity (**B**), and APD50 (**C**) after 24 h of treatment in PA, PA + EMPA and control group. n = 6 per group. ns *p* ＞ 0.05, * *p* < 0.05, ** *p* < 0.01.


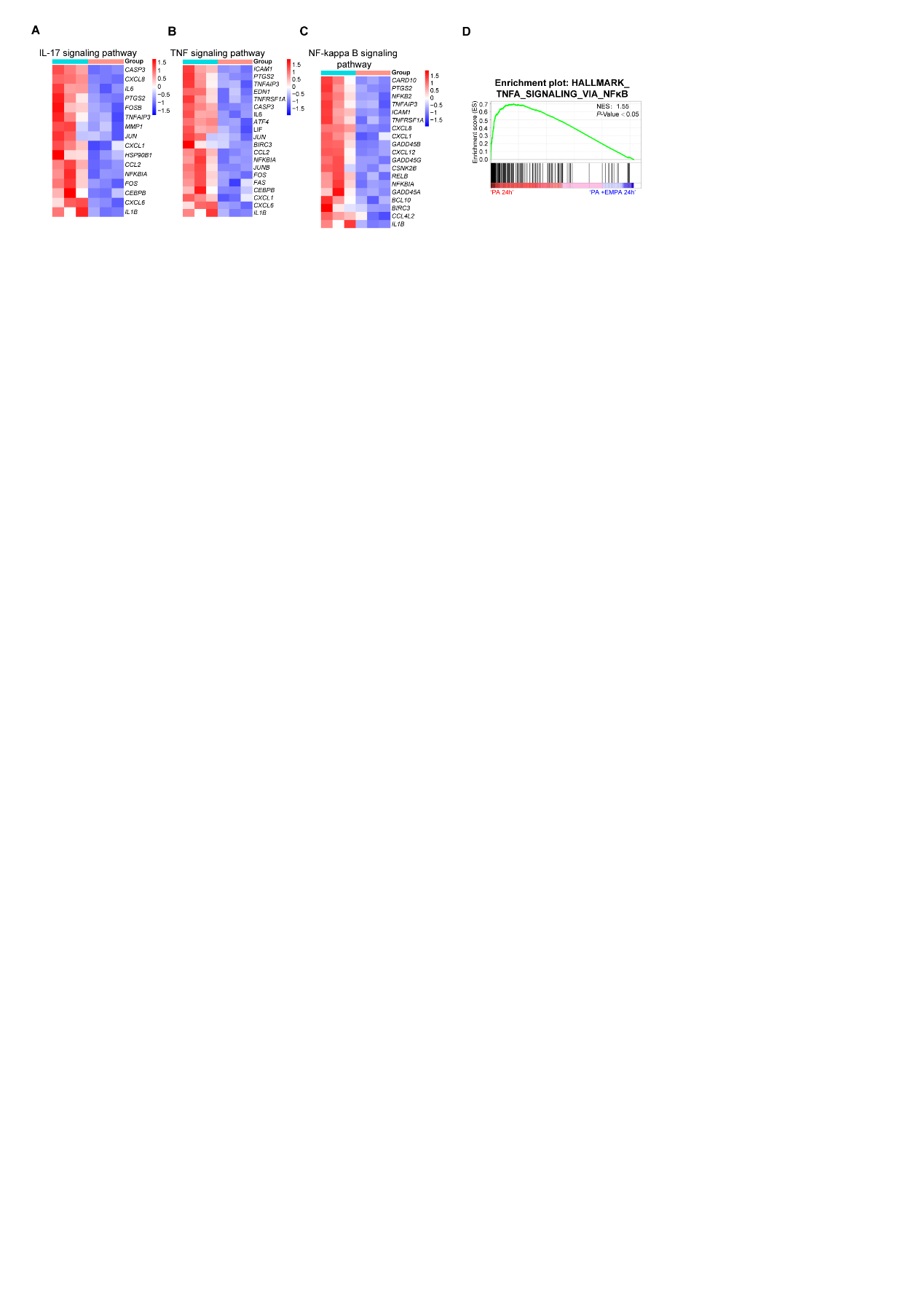


**Supplementary Figure. S8: Inflammatory signaling pathways are suppressed in EMPA-treated PA-induced hEHT models by RNA sequencing.** (**A**-**C**) Heatmaps showing the downregulation of inflammatory pathways including IL-17 signaling pathway (**A**), TNF signaling pathway (**B**), and NF-κB signaling pathway (**C**) in the PA+EMPA group compared to the PA group. (**D**) GSEA showed enrichment of TNF signaling pathway in the PA group relative to the PA+EMPA group.


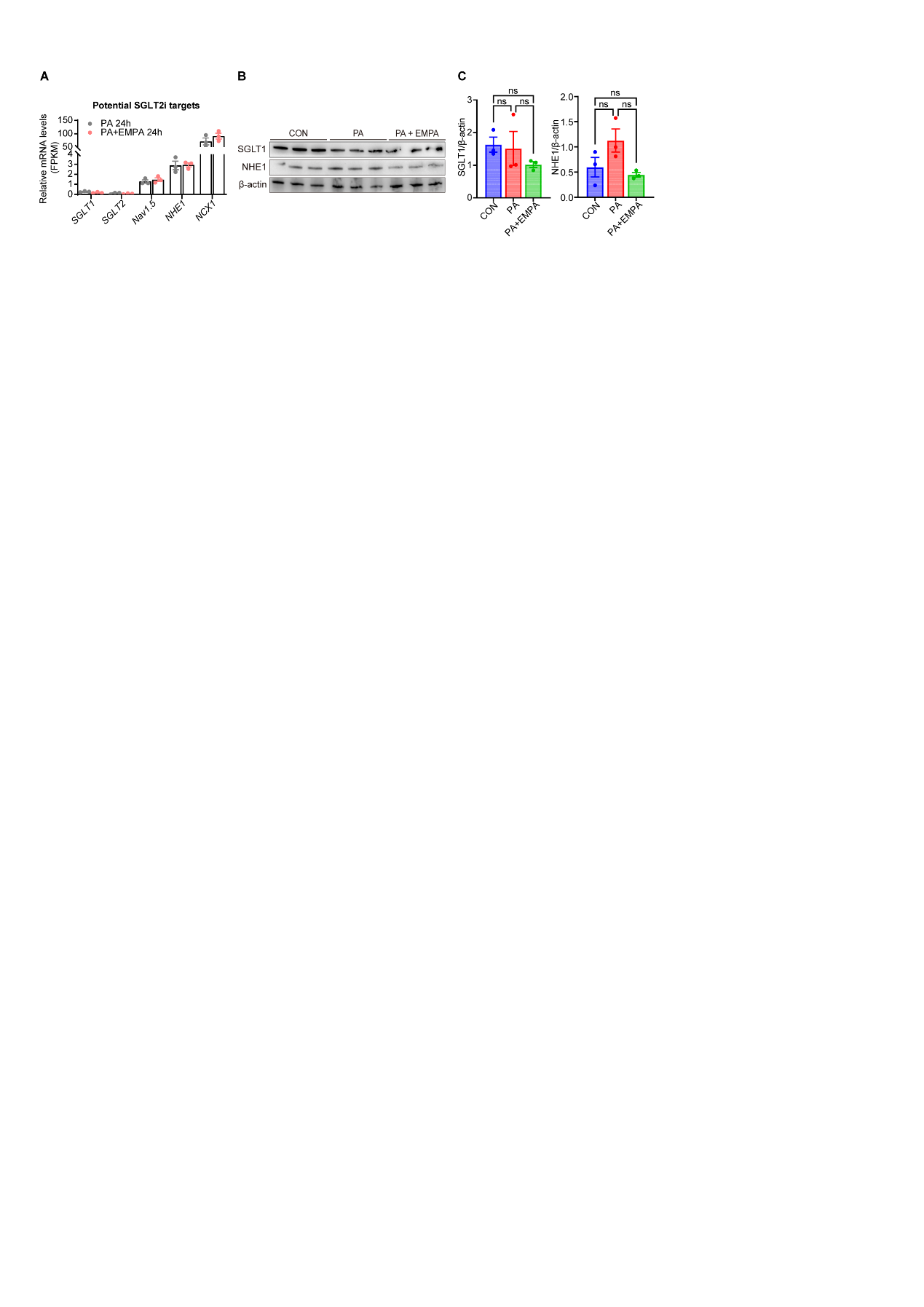


**Supplementary Figure. S9: The effect of EMPA on potential SGLT2 inhibitor targets in PA-treated hEHTs after 1 day.** (**A**) FPKM values from RNA sequencing of hEHTs treated with PA or PA+EMPA for 24 hours. (**B**-**C**) Representative immunoblot (B) and quantification (C) of SGLT1 and NHE1 protein levels in hEHTs treated with control (CON), PA, or PA+EMPA for 24 hours. β-Actin served as the loading control. n = 3 biological replicates. ns *p* ＞ 0.05.
